# Orthopaedic Surgery Didactic Session Improves Confidence in Distal Radius Fracture Management by Emergency Medicine Residents

**DOI:** 10.21980/J8K365

**Published:** 2025-04-30

**Authors:** Ian T Watkins, Jessica L Duggan, Aron Lechtig, Andrew Bauder, Luke He, Alexy Ilchuk, Amanda Doodlesack, Carl Harper, Tamara D Rozental

**Affiliations:** * Harvard Combined Orthopaedic Residency Program, Boston, Massachusetts; ^ Beth Israel Deaconess Medical Center, Division of Hand and Upper Extremity Surgery, Boston, Massachusetts; † Harvard Medical School, Boston, Massachusetts; ** Beth Israel Deaconess Medical Center, Department of Emergency Medicine, Boston, Massachusetts

## Abstract

**Audience:**

This didactic session on distal radius fracture diagnosis and management is designed for Emergency Medicine (EM) residents of all levels.

**Introduction:**

With an incidence of 1,130 upper extremity injuries per 100,000 persons per year,^1^ distal radius fractures (DRFs) are the most common adult fracture, representing 17.5% of all fractures.^2^–^4^ Yet, many emergency medicine residents feel unprepared to manage DRFs independently upon graduation.^5^ The standard management of a fracture in the ED setting consists of identifying any urgent aspects of the fracture, controlling pain, performing a reduction if necessary, and applying a splint.^6^ Poor reduction or splinting techniques can lead to serious complications, including acute carpal tunnel or compartment syndrome, development of severe burns and rarely, amputation.^6^–^8^ Though it is common for emergency medicine (EM) resident trainees working in academic institutions to have regular access to orthopaedic surgery consultation, many will go on to practice in community settings or departments without access to full-time orthopaedic coverage. It is essential for EM residents to be familiar with DRF diagnosis and management, including closed reduction and splinting. We seek to create a toolbox for managing upper extremity fractures, with the overall purpose of improving orthopaedic care in the ED setting.

**Educational Objectives:**

By the end of this didactic session, learners should be able to: 1) assess DRF displacement on pre-reduction radiography and formulate reduction strategies, 2) perform a closed reduction of a DRF, 3) apply a safe and appropriate plaster splint to patient with a DRF and assess the patient’s neurovascular status, 4) assess DRF post-reduction radiography for relative fracture alignment, and 5) understand appropriate follow-up and necessary return precautions.

**Educational Methods:**

Learners attended a didactic session led by orthopaedic surgery residents which included a faculty-approved lecture on DRFs and hands-on skills workshop on reducing the fractures and effectively applying plaster splints.

**Research Methods:**

Prior to the educational session, participants completed a pre-workshop survey assessing current practices and baseline confidence regarding DRF management. Self-confidence levels for each skill were measured using a Likert scale from 0 (least confident) to 100 (most confident). Confidence levels were re-assessed immediately after the didactic session and three months later.

**Results:**

Nineteen emergency medicine (EM) residents (n=12, 63% female) across three class years (n=9, 47% PGY 1; n=6, 32% PGY 2; n=4, 21% PGY 3) completed the pre-workshop survey, and 15 residents participated in the didactic session and completed follow-up surveys. Fourteen (75%) EM residents reported reducing DRFs on their own (without an orthopaedic consult) less than half of the time. After the workshop, confidence levels increased significantly across all seven domains of DRF management, most notably in applying a plaster splint (+31.9 points, p<0.001), teaching DRF splinting techniques (+37.0 points, p<0.001), and managing DRF care in the ED independently (+34.6 points, p<0.001). These improvements persisted three months later.

**Discussion:**

The didactic session and skills workshop on DRF management were effective in improving EM residents’ confidence measures in the short term. The session was well-received by the residents, who unanimously expressed interest in collaboration for future orthopaedic workshops. Further work should replicate this study with a larger sample and develop skills assessments to objectively evaluate learners’ abilities in the short and long-term.

**Topics:**

Distal radius fracture, reduction, splinting, collaboration, orthopaedic surgery, orthopaedics, resident education.

## USER GUIDE

**Table:** 

List of Resources:	
Abstract	1
User Guide	3
Small Groups Learning Materials	9
Appendix A: Distal Radius Fractures Slides	9

**Learner Audience:**

Interns, Junior Residents, Senior Residents

**Time Required for Implementation:**

3 hours

**Recommended Number of Learners per Instructor:**

10 learners

**Topics:**

Distal radius fracture, reduction, splinting, collaboration, orthopaedic surgery, orthopaedics, resident education.

**Objectives:**

By the end of this didactic session, learners should be able to:

1. Assess DRF displacement on pre-reduction radiography and formulate reduction strategies.
2. Perform a closed reduction of a DRF.
3. Apply a safe and appropriate plaster splint to patient with a DRF and assess the patient’s neurovascular status.
4. Assess DRF post-reduction radiography for relative fracture alignment.
5. Understand appropriate follow-up and necessary return precautions.

### Linked objectives and methods

The three-hour didactic session consisted of two components: a lecture on approach to DRFs in the ED as well as a hands-on skills workshop in fracture reduction and plaster splint application. We chose to incorporate both modalities because it is important to have a background understanding of this fracture pattern and patient presentation prior to practicing the hands-on reduction techniques. This training session was led by two orthopaedic surgery residents who were trained by senior faculty. Educational materials were designed by senior faculty based on the American Society for Surgery of the Hand and American Academy of Orthopaedic Surgeons core curricula.^9^,^10^

The lecture included epidemiology of DRFs and patient presentation, anatomy of the wrist joint, radiographic interpretation (Objectives 1,5), and the patient management timeline (Objective 6). Management topics included placing a hematoma block for pain control (Objective 2), fracture reduction, splinting, and follow-up decision making. We emphasized several patient safety measures: identifying open fractures, timely administration of antibiotics, diagnosing acute carpal tunnel/compartment syndrome, and applying safe and appropriate splints. (See attached PowerPoint document).

The skills workshop focused on safe and reliable reduction maneuvers and application of plaster splints. We first reviewed how to place the patient in traction using a finger trap with 4.5-inch rolled gauze. Next, we reviewed how to prepare/roll out plaster splints and set up all equipment required for splinting. We then practiced the technique for reducing a distal radius fracture and using a three-point mold after applying the plaster splint to maintain the reduction (Objectives 3,4). All learners were given the chance to practice these techniques under supervision of the instructors. (See Small Group Stations).

### Recommended pre-reading for facilitator

1. Padegimas EM, Ilyas AM. Distal radius fractures: emergency department evaluation and management. *Orthop Clin North Am.* 2015;46(2):259–270. https://doi.org/10.1016/j.ocl.2014.11.010
2. Chhabra AB, Yildirim B. Adult distal radius fracture management. *JAAOS.* 2021;29(22):e1105–e1116. https://doi.org/10.5435/JAAOS-D-20-01335
3. Roque PJ, Barker B, Gridley D, Stapczynski JS, LoVecchio F. 213 Use of radiographic parameters for distal radius fracture reduction analysis. *An Emerg Med*. 2011;58(4):S249. https://doi/org/10.1016/j.annemergmed.2011.06.242
4. Jorgensen A, Kahan J, Moran J, Halim A. Assessment of complications associated with casting of acute distal radius fractures in adults. *Am J Emerg Med.* 2022;56:124–126. https://doi.org/10.1016/j.ajem.2022.03.017

### Learner responsible content (LRC)

1. Padegimas EM, Ilyas AM. Distal radius fractures: emergency department evaluation and management. *Orthop Clin North Am.* 2015;46(2):259–270. https://doi.org/10.1016/j.ocl.2014.11.010
2. Chhabra AB, Yildirim B. Adult distal radius fracture management. *JAAOS.* 2021;29(22):e1105–e1116. https://doi.org/10.5435/JAAOS-D-20-01335

### Large group lecture (1 hour)

Distal Radius Fracture Slides

### Hands-on Stations (2 hours)

#### Materials per 1 group of 4 learners (photos embedded within PowerPoint lecture)

- 2× 4.5-inch rolled gauze (for finger trap)
- 2× weights or 6x 1-Liter intravenous fluids bags in stockinette
- 4× rolls of soft undercast padding (3” or 4”)
- 4× rolls of plaster (4”)
- 4× soft elastic bandage wrap (3” or 4”)
- 1× plastic basin with warm water
- 1× silk tape
- 1× scissors

#### Instructions

1. Divide students into small groups (3–5 students per group), with one faculty member/orthopaedic resident instructor per group. Learners will take turns being the patient and the clinician.
2. Allow learners to practice hanging the “patient’s” arm in traction using the finger trap method with 4.5-inch rolled gauze and intravenous fluid bags as counterweight.
3. Demonstrate and have learners practice maneuvers for reduction of a dorsally displaced distal radius fracture.
4. Ensure all learners can perform an adequate upper extremity neurovascular exam.
5. Demonstrate how to set up splinting material: roll out two splints (dorsal and volar) of 6 layers of soft cast padding and 10 layers of plaster each. When ready, dip plaster in warm water basin and press out excess water. Ensure the soft padded side of the splint is in contact with the patient’s skin because plaster will become hot and can cause burns.
6. Have learners practice applying splint with proper three-point mold. Then apply elastic bandage wrap/tape and re-assess the patient’s neurovascular status.

### Results and tips for successful implementation

#### Participants

Nineteen out of 39 residents (49%) completed the pre-workshop survey (Table 1). Junior residents comprised most of the participants (n=9, 47% PGY 1 and n=6, 32% PGY 2) when compared to senior residents (n=4, 21% PGY 3). Four of the 19 residents who completed the pre-workshop survey were unable to attend the workshop due to clinical duties or illness; their responses were included in all pre-workshop results. The remaining 15 residents who attended the workshop all completed the post-workshop and three-month follow-up surveys.

#### Current Practices

A large majority of EM residents (n=14, 74%) indicated that they often rely on a bedside orthopaedic consultation to reduce closed DRFs, performing reductions on their own less than half of the time. Yet fourteen (74%) also reported that they expect to independently reduce closed DRFs more than half of the time as attendings. The greatest barriers to reducing DRFs were time pressures in the ED and acuity of other patients (reported by 90% of residents, n=17), followed by pressure from attendings to consult orthopaedics (reported by 58%, n=11). In terms of the fracture characteristics, a DRF that is “open” was the most common factor inclining participants (n=18, 100%) to consult orthopaedics, followed by both radius and ulna fractures (n=13, 72%), intra-articular involvement (n=12, 67%), and the presence of comminution (n=10, 56%).

#### Improvement in Confidence

Self-confidence levels were measured using a Likert scale from 0 (least confident) to 100 (most confident). When compared to pre-workshop surveys (Table 2), self-reported confidence and preparedness levels increased significantly across all seven sub-categories of DRF management following the orthopaedic workshop (Table 3). The most notable improvements were confidence in applying a plaster splint (+31.9 points, p<0.001), teaching DRF splinting techniques (+37.0 points, p<0.001), and managing DRF care in the ED independently (+34.6 points, p<0.001). The effect sizes of these measures were all large, with a Cohen’s d of 1.26, 1.66, and 1.74, respectively. The remaining categories were confidence in interpreting DRF radiography pre- and post-reduction (+12.4, p=0.02 and +22.1, p=0.001, respectively), providing analgesia during reductions (+10.3, p=0.007), and effectively reducing a DRF (+20.0, p<0.001).

When stratified into junior (PGY 1 and PGY 2) and senior (PGY 3) residents, there were no significant group differences.

#### Post-Workshop

Following the workshop, the mean rating for the didactic session being a valuable use of time was 97.7±5.6 out of 100, and the mean rating for desire for more orthopaedic-oriented didactic sessions was 97.7±6.2 (Table 4). Over half of participants rated their preparation to independently manage DRFs as 80/100 and above, and only one quarter of participants rated this item below 70/100. The vast majority indicated that DRF management will be an important part of their practice as attending ED physicians (mean rating 89.0±14.6).

#### Three-Month Follow-up

At the three-month time point, there were no significant differences in any of the seven confidence measures when compared to the post-workshop results (Table 5). The perceived importance of reduction and splinting for EM doctors increased slightly (+4.7, p=0.03) at three months. Participants continued to rate the didactic session as a valuable use of time (97.1±6.4) and expressed interest in having more orthopaedic-oriented didactic sessions (97.9±5.0), and these variables remained unchanged from the post-workshop results. None of the 15 workshop participants were lost to follow-up.

#### Implementation and Improvement

For the didactic session to run efficiently, the presenters (if multiple) should prepare ahead of time how they will teach the lecture portion and agree upon the level of detail they hope to achieve. A timekeeper should be assigned to ensure there is sufficient time for the hands-on portion. The presenters should then split up, such that there is at least one orthopaedic surgery team member in each skills group to review reduction and splinting techniques. While our didactic was well-received by the residents overall, there are several areas for improvement. First, an objective knowledge and skills examination may help to measure learners’ baseline abilities and improvement over time, as opposed to solely evaluating confidence levels. Second, the session could be modified into a more advanced module targeted at senior residents who likely have more experience managing DRFs.

### Associated Content

Appendix A: Distal Radius Fractures PowerPoint Slides

### Pearls

- Distal radius fractures (DRFs) are the most common adult fracture, representing 17.5% of all fractures. The incidence of these fractures continues to increase as the population over age 65 increases.^2^–^4^
- Consider the following factors when you have a patient first present to you with a DRF:
  ○ Is the fracture open? If so, start antibiotics (1^st^ generation cephalosporin) as soon as possible.
  ○ Have you ordered the proper radiographs? Review the degree of fracture displacement and assess for concurrent injuries.
    ■ Assess for atypical fracture patterns, including: volar displacement of the distal fragment, significant comminution, and other injury patterns that may require different approaches.
  ○ What is the patient’s neurovascular status on presentation? Make sure to assess for acute carpal tunnel syndrome.
  ○ Have a plan for pain control (oral, intravenous, hematoma block).
- Prior to reduction, set up a finger trap to allow for traction at the fracture site for 5–10 minutes while you obtain/set-up splinting materials.
- Use radiographs to guide a safe and successful reduction.
  ○ Use injury radiographs to guide your reduction maneuvers. Understand that volar displaced DRFs are managed differently than the standard dorsally displaced DRFs.
  ○ Perform reduction by re-creating the deformity, re-establishing length, and restoring alignment.
- Apply a plaster splint using a three-point mold and hold in position until plaster has hardened.
- Assess post-reduction radiographs, understanding what constitutes an acceptable result.
- Be aware of serious complications, including neurovascular injury, burns, and compartment syndrome.
- Remember to establish a plan for adequate follow-up.
- Recommended references to skim (see further reading for additional resources):
  ○ Padegimas EM, Ilyas AM. Distal radius fractures: emergency department evaluation and management. *Orthop Clin North Am.* 2015;46(2):259–270. doi: 10.1016/j.ocl.2014.11.010
  ○ Roque PJ, Barker B, Gridley D, Stapczynski JS, LoVecchio F. 213 Use of radiographic parameters for distal radius fracture reduction analysis. *Ann Emerg Med.* 2011;58(4):S249. doi: 10.1016/j.annemergmed.2011.06.242

## Supplementary Information



## Figures and Tables

**Table 1:** Baseline demographics and anticipated practice settings of the study participants.

Factors		Respondents (N=19)
**Level of Training**		
	PGY-1	9 (47.4%)
	PGY-2	6 (31.6%)
	PGY-3	4 (21.0%)
**Gender**		
	Female	12 (63.2%)
	Male	7 (36.8%)
**Anticipated Practice Setting**		
	Large academic tertiary care center	4 (21.0%)
	Non-tertiary care center with basic residency programs	6 (31.6%)
	Community hospital with few-to-no residency programs	4 (21.0%)
	Hospital administrative setting / Non-clinical	0 (0.0%)
	Not sure	5 (26.3%)
	Other not listed	0 (0.0%)
**Anticipated Orthopedic Coverage**		
	Yes	5 (26.3%)
	No	4 (21.0%)
	Uncertain	10 (52.6%)

**Table 2:** Pre-workshop DRF management confidence ratings.

Survey Item	Min	Median	Max	Mean	SD
I am confident in interpreting DRF radiography.	8	63	95	57.74	24.54
I am confident in providing analgesia during reductions.	20	75	100	69.00	23.12
I am confident in effectively reducing a DRF.	0	57.5	80	51.22	25.89
I am confident in applying a plaster splint.	0	50	100	48.28	29.2
I am confident in assessing post reduction radiographs.	11	57	90	51.44	23.38
I am confident in teaching DRF splinting techniques to other trainees.	0	30	66	31.47	23.09
It is important for an EM doctors to be able to reduce and splint DRFs.	50	100	100	90.21	14.28
Reduction and splinting DRFs will be important part of practice as an attending.	30	87	100	80.84	21.24
There is no point in reducing DRF if it will be managed surgically.	0	20	28	13.42	10.93
All DRFs that I have seen in the ED are eventually managed surgically.	0	18	50	23.41	21.97
Proper splinting after reduction may lead to non-operative management.	50	81	100	81.17	14.61
I feel prepared to independently manage DRF care in the ED.	0	44	75	38.50	23.8

**Table 3:** Changes in DRF management confidence before and after orthopedic-led lecture and workshop.

Survey Item	Mean Pre-Workshop	Mean Post-Workshop	Difference in Means	P-value (alpha 0.05)	Effect Size (Cohen’s d)
I am confident in interpreting DRF radiography.*	57.74	70.13	12.39	0.016	0.679
I am confident in providing analgesia during reductions.*	69.00	79.31	10.31	0.007	0.788
I am confident in effectively reducing a DRF.*	51.22	71.20	19.98	<0.001	1.169
I am confident in applying a plaster splint.*	48.28	80.13	31.85	<0.001	1.262
I am confident in assessing post reduction radiographs.*	51.44	73.53	22.09	0.001	1.033
I am confident in teaching DRF splinting techniques to other trainees.*	31.47	68.47	37.00	<0.001	1.656
It is important for ED doctors to be able to reduce and splint DRFs.	90.21	94.00	3.79	0.089	0.455
Reduction and splinting DRFs will be important part of practice as an attending.*	80.84	89.00	8.16	0.035	0.581
There is no point in reducing DRF if it will be managed surgically.	13.42	7.93	−5.49	0.061	−0.506
All DRFs that I have seen in the ED are eventually managed surgically.	23.41	23.60	0.19	0.658	0.113
Proper splinting after reduction may lead to non-operative management.	81.17	87.00	5.83	0.368	0.220
I feel prepared to independently manage DRF care in the ED.*	38.50	73.07	34.57	<0.001	1.735

* Statistically significant at alpha level 0.05 with paired samples t-test (two-tailed)

**Table 4:** Post-workshop survey with DRF management confidence ratings.

Survey Item	Min	Median	Max	Mean	SD
I am confident in interpreting DRF radiography.	50	70	90	70.13	14.07
I am confident in providing analgesia during reductions.	60	80	100	79.31	11.72
I am confident in effectively reducing a DRF.	45	71	93	71.20	16.24
I am confident in applying a plaster splint.	60	80	100	80.13	12.59
I am confident in assessing post reduction radiographs.	50	75	100	73.53	14.89
I am confident in teaching DRF splinting techniques to other trainees.	26	75	90	68.47	18.87
It is important for an EM doctors to be able to reduce and splint DRFs.	80	100	100	94.00	7.25
Reduction and splinting DRFs will be important part of practice as an attending.	50	94	100	89.00	14.61
There is no point in reducing DRF if it will be managed surgically.	0	0	29	7.93	11.18
All DRFs that I have seen in the ED are eventually managed surgically.	0	23	57	23.60	24.64
Proper splinting after reduction may lead to non-operative management.	70	90	100	87.00	12.86
This training was valuable use of your time.	80	100	100	97.67	5.63
I would like to have more ortho-oriented didactics.	80	100	100	97.67	6.23
I feel prepared to independently manage DRF care in the ED.	39	80	88	73.07	14.48

**Table 5:** Three-month follow-up survey with DRF management confidence ratings.

Survey Item	Min	Median	Max	Mean	SD
I am confident in interpreting DRF radiography.	45	75	100	73.20	14.29
I am confident in providing analgesia during reductions.	35	80	100	77.93	15.65
I am confident in effectively reducing a DRF.	57	70	100	70.27	11.67
I am confident in applying a plaster splint.	55	75	100	76.2	12.95
I am confident in assessing post reduction radiographs.	50	75	100	78.07	14.37
I am confident in teaching DRF splinting techniques to other trainees.	40	65	80	63.27	14.08
It is important for an EM doctor to be able to reduce and splint DRFs.	90	100	100	98.67	3.52
Reduction and splinting DRFs will be important part of practice as an attending.	72	100	100	93.87	9.73
There is no point in reducing DRF if it will be managed surgically.	0	10	40	12.40	13.04
All DRFs that I have seen in the ED are eventually managed surgically.	0	20	80	28.67	24.16
Proper splinting after reduction may lead to non-operative management.	0	85	100	79.67	26.16
This training was valuable use of your time.	80	100	100	97.07	6.36
I would like to have more ortho-oriented didactics.	83	100	100	97.87	4.97
I feel prepared to independently manage DRF care in the ED.	35	73	100	72.33	19.79

**Figure:**
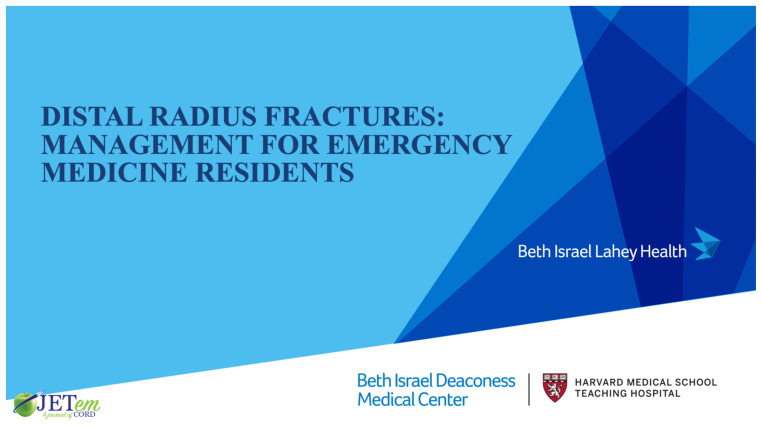
Please see associated PowerPoint file
