# Supplementary material for: Orthopaedic Surgery Didactic Session Improves Confidence in Distal Radius Fracture Management by Emergency Medicine Residents
Source: J Educ Teach Emerg Med. 2025 Apr 30;10(2):SG1–9. doi: 10.21980/J8K365 (PMC12054089; doi:10.21980/J8K365)
Supplement: Supplementary file 1 [file 10-2-SG1-supp1.pptx]

## Slide 1
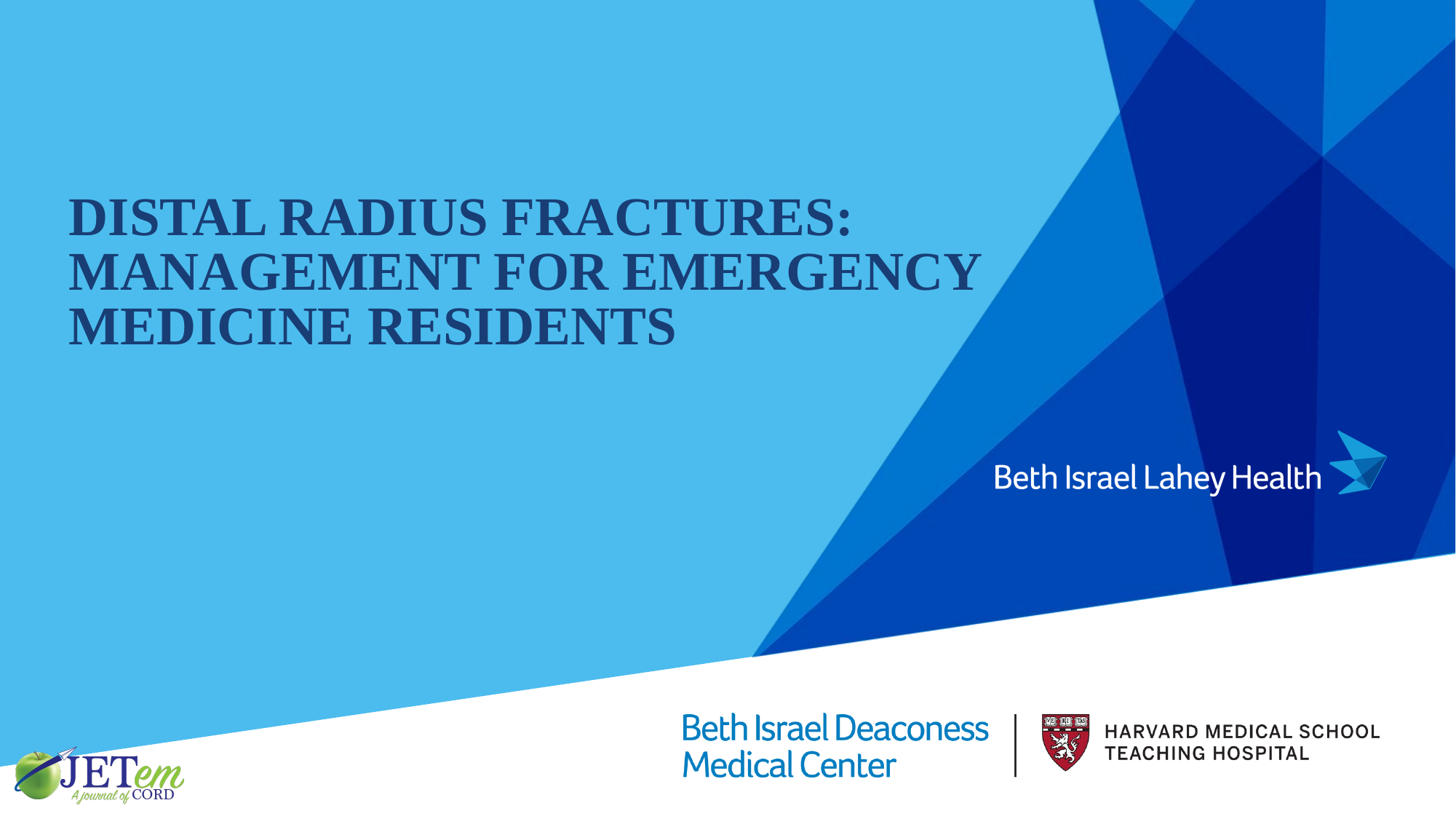

# Distal Radius fractures: Management for emergency medicine residents

## Slide 2
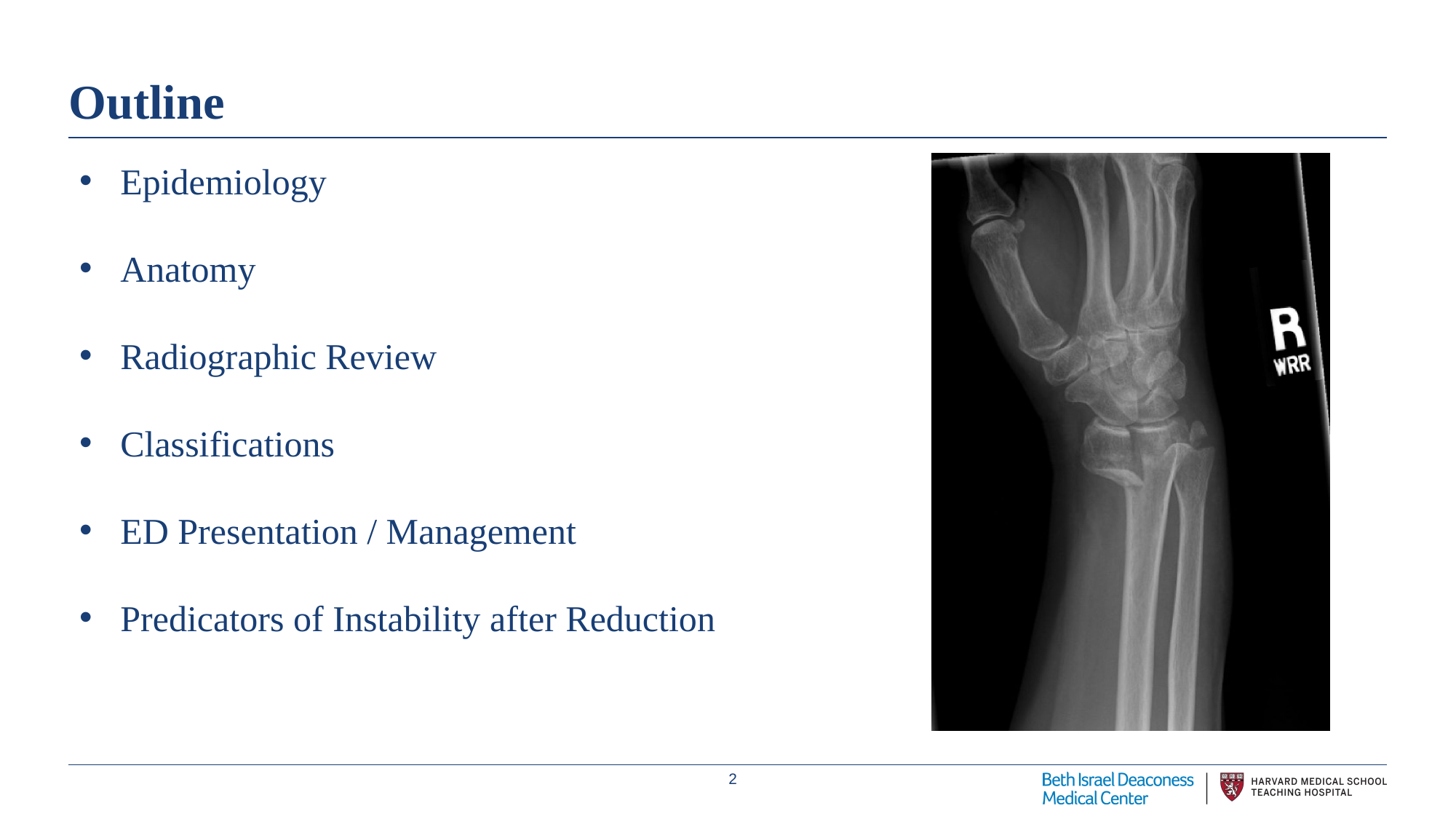

# Outline
Epidemiology
Anatomy
Radiographic Review
Classifications
ED Presentation / Management
Predicators of Instability after Reduction
2

## Slide 3
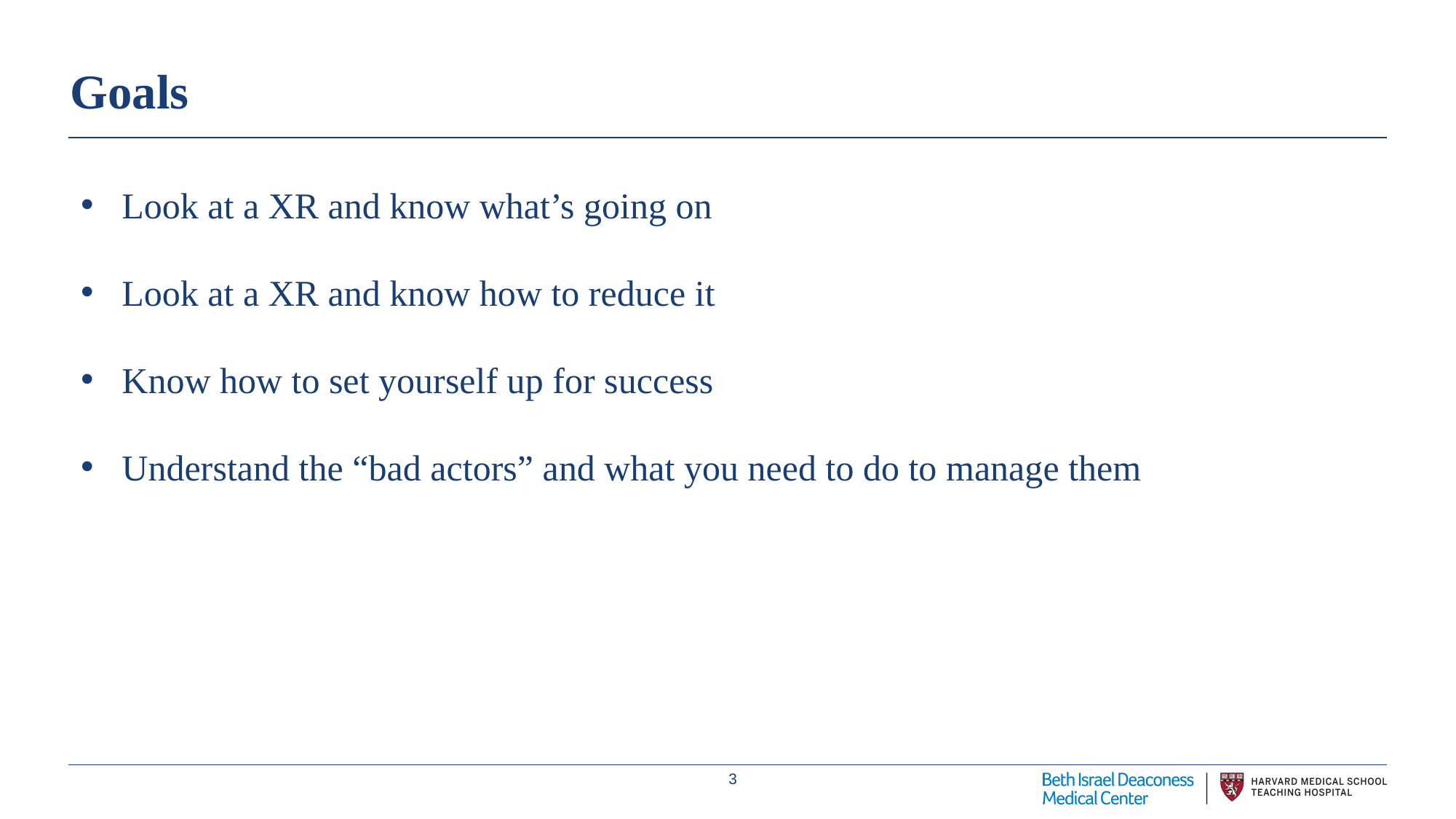

# Goals
Look at a XR and know what’s going on
Look at a XR and know how to reduce it
Know how to set yourself up for success
Understand the “bad actors” and what you need to do to manage them
3

## Slide 4
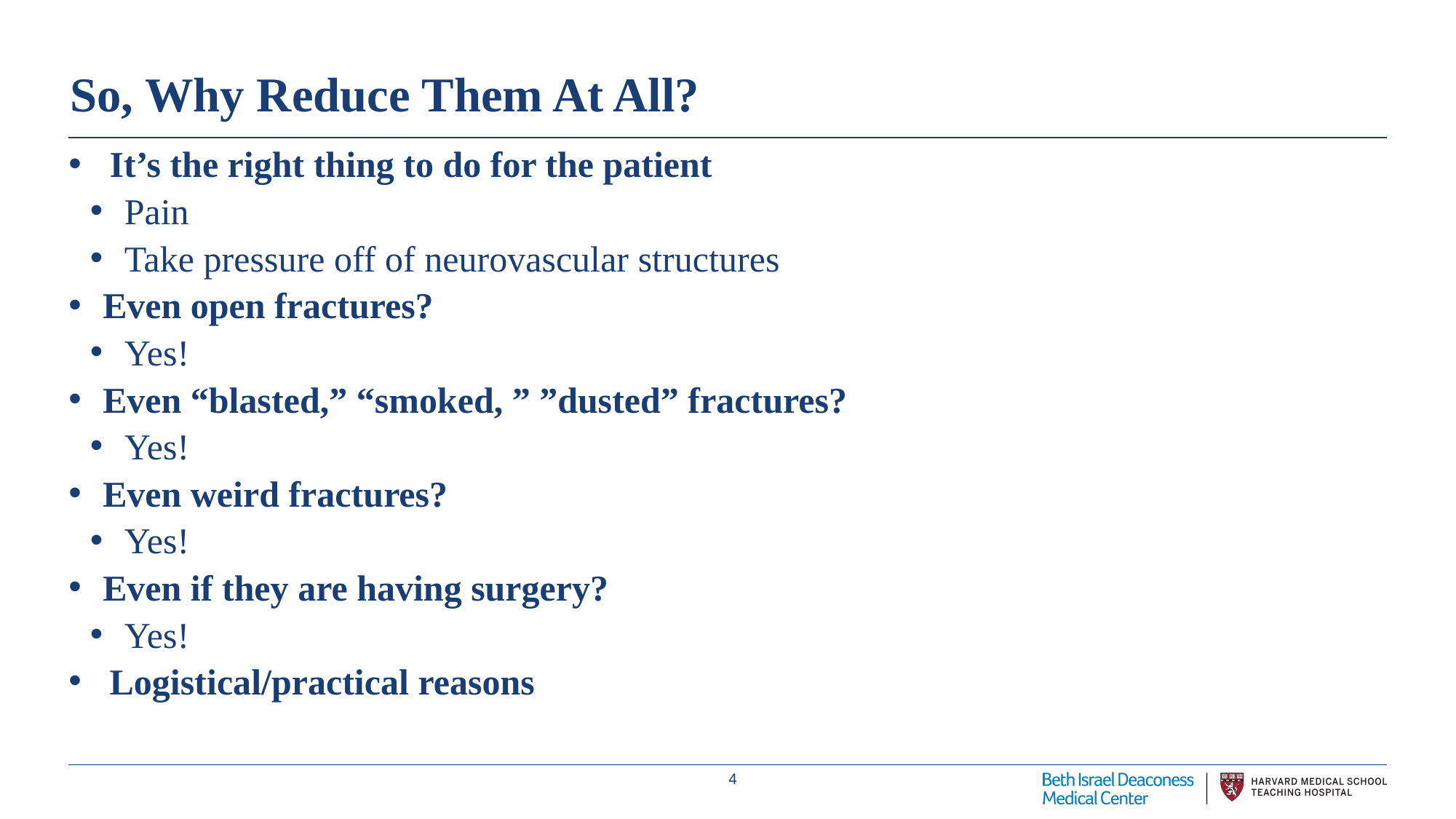

# So, Why Reduce Them At All?
It’s the right thing to do for the patient
Pain
Take pressure off of neurovascular structures
Even open fractures?
Yes!
Even “blasted,” “smoked, ” ”dusted” fractures?
Yes!
Even weird fractures?
Yes!
Even if they are having surgery?
Yes!
Logistical/practical reasons

## Slide 5
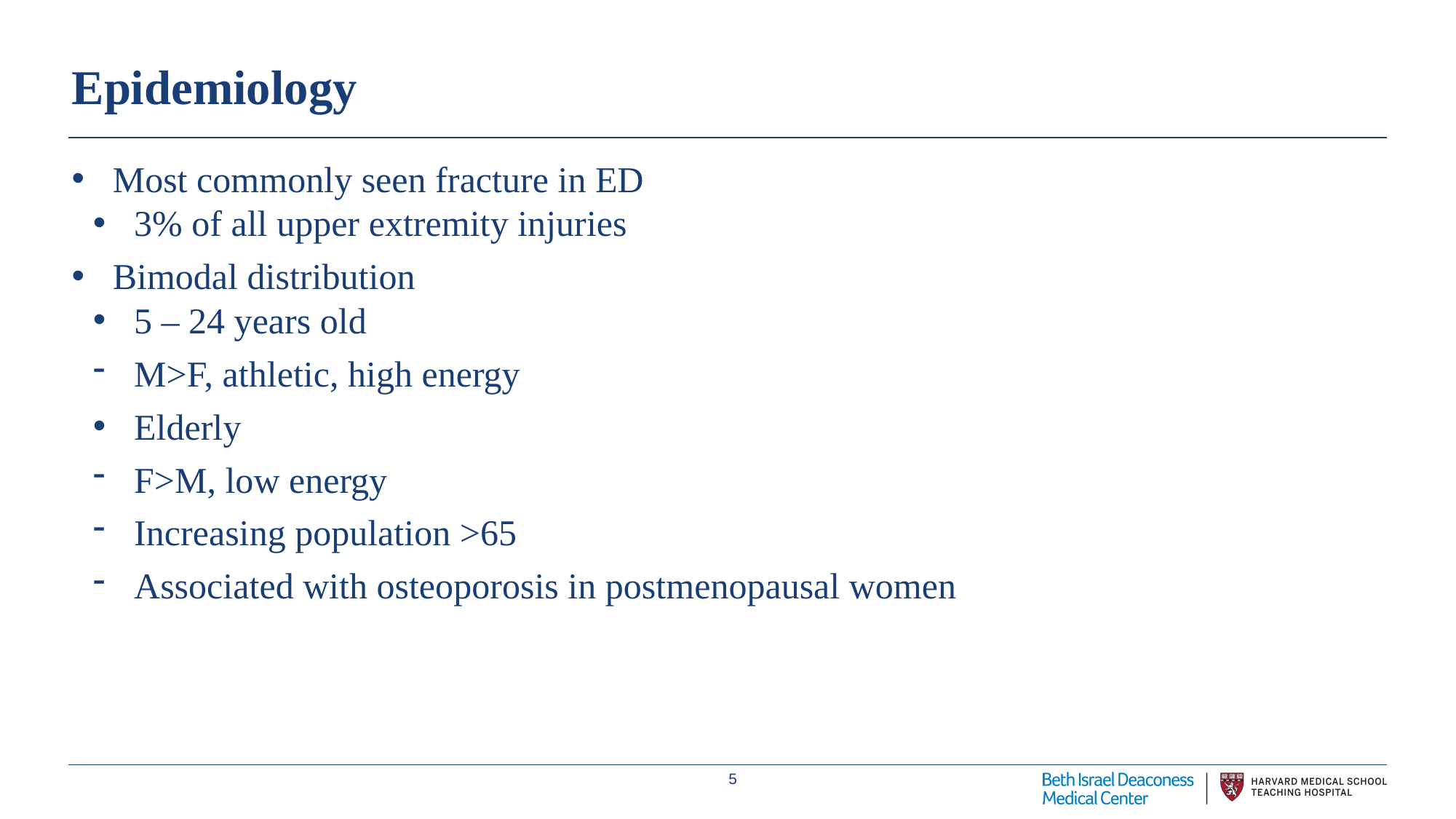

# Epidemiology
Most commonly seen fracture in ED
3% of all upper extremity injuries
Bimodal distribution
5 – 24 years old
M>F, athletic, high energy
Elderly
F>M, low energy
Increasing population >65
Associated with osteoporosis in postmenopausal women
5

## Slide 6
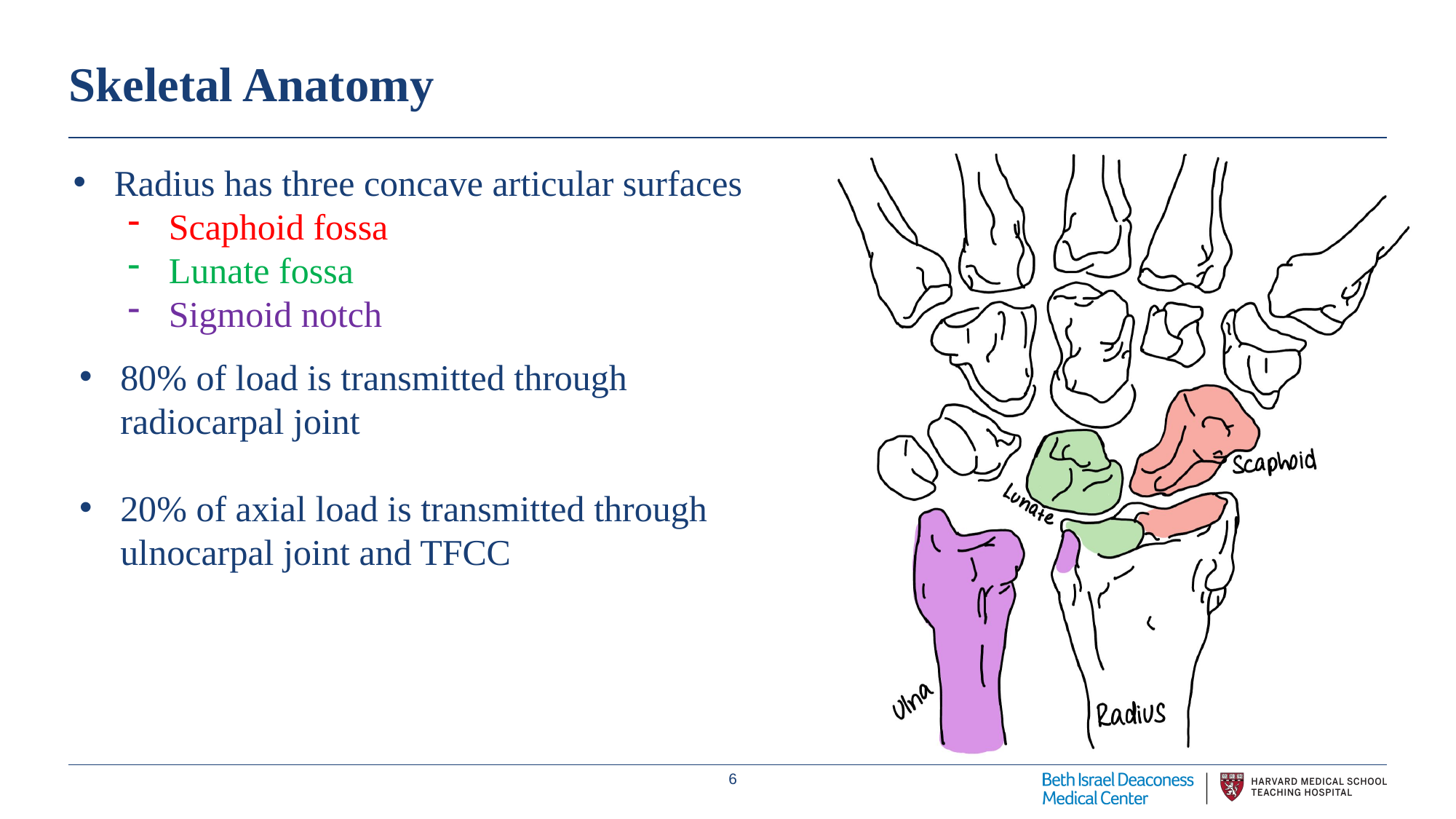

# Skeletal Anatomy
Radius has three concave articular surfaces
Scaphoid fossa
Lunate fossa
Sigmoid notch
80% of load is transmitted through radiocarpal joint
20% of axial load is transmitted through ulnocarpal joint and TFCC

## Slide 7
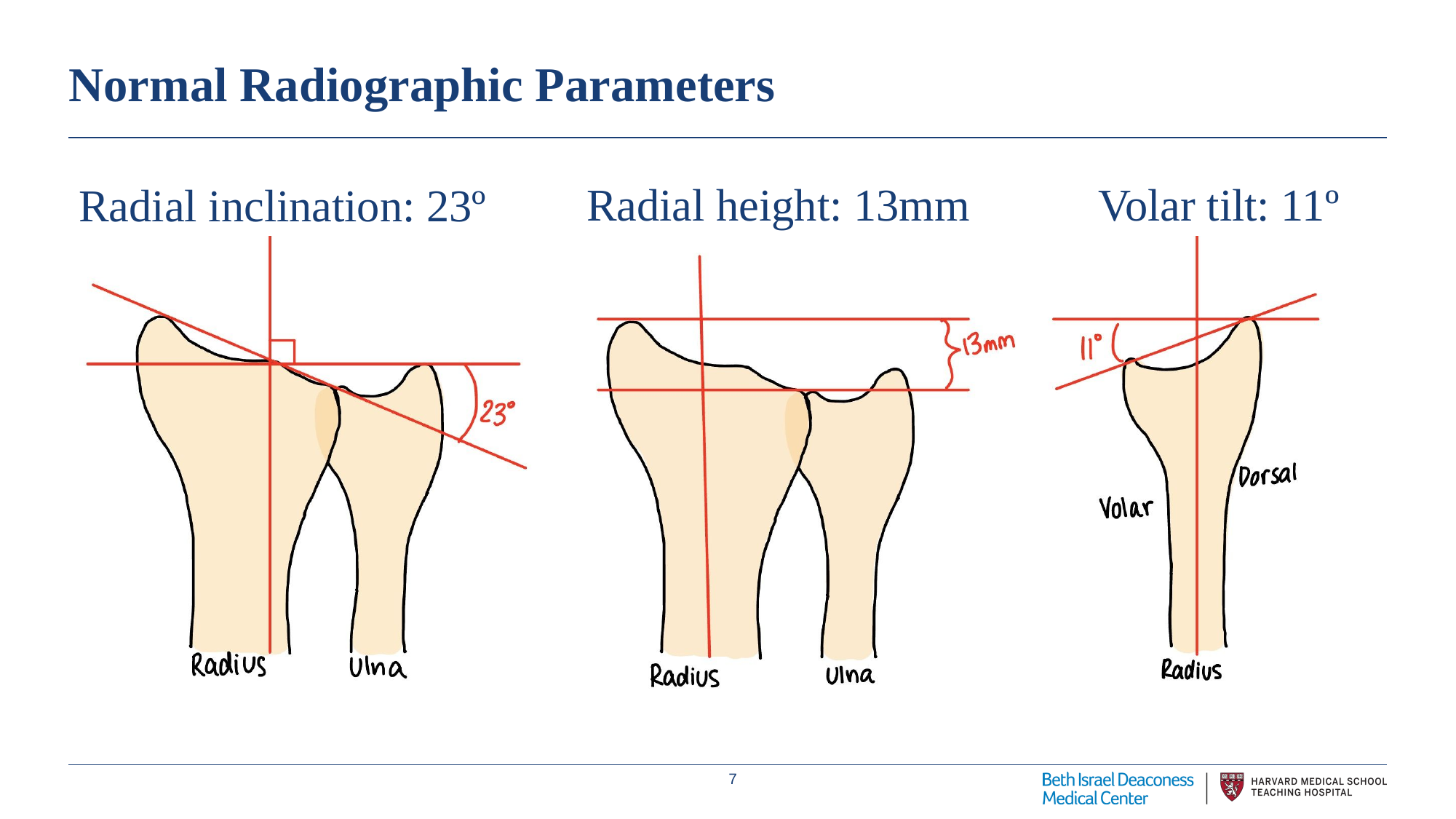

# Normal Radiographic Parameters
Radial height: 13mm
Volar tilt: 11º
Radial inclination: 23º

## Slide 8
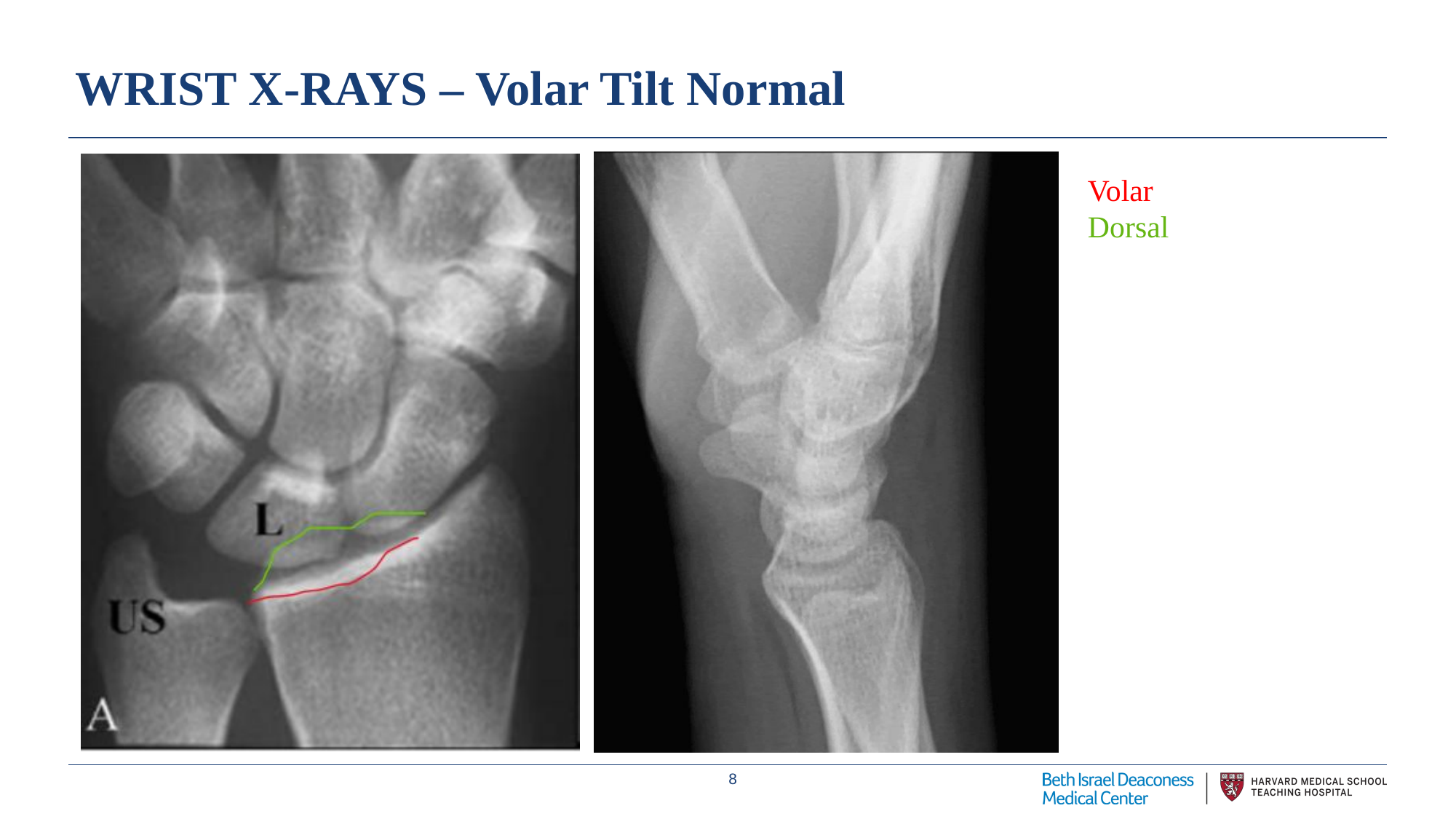

WRIST X-RAYS – Volar Tilt Normal
Volar
Dorsal

## Slide 9
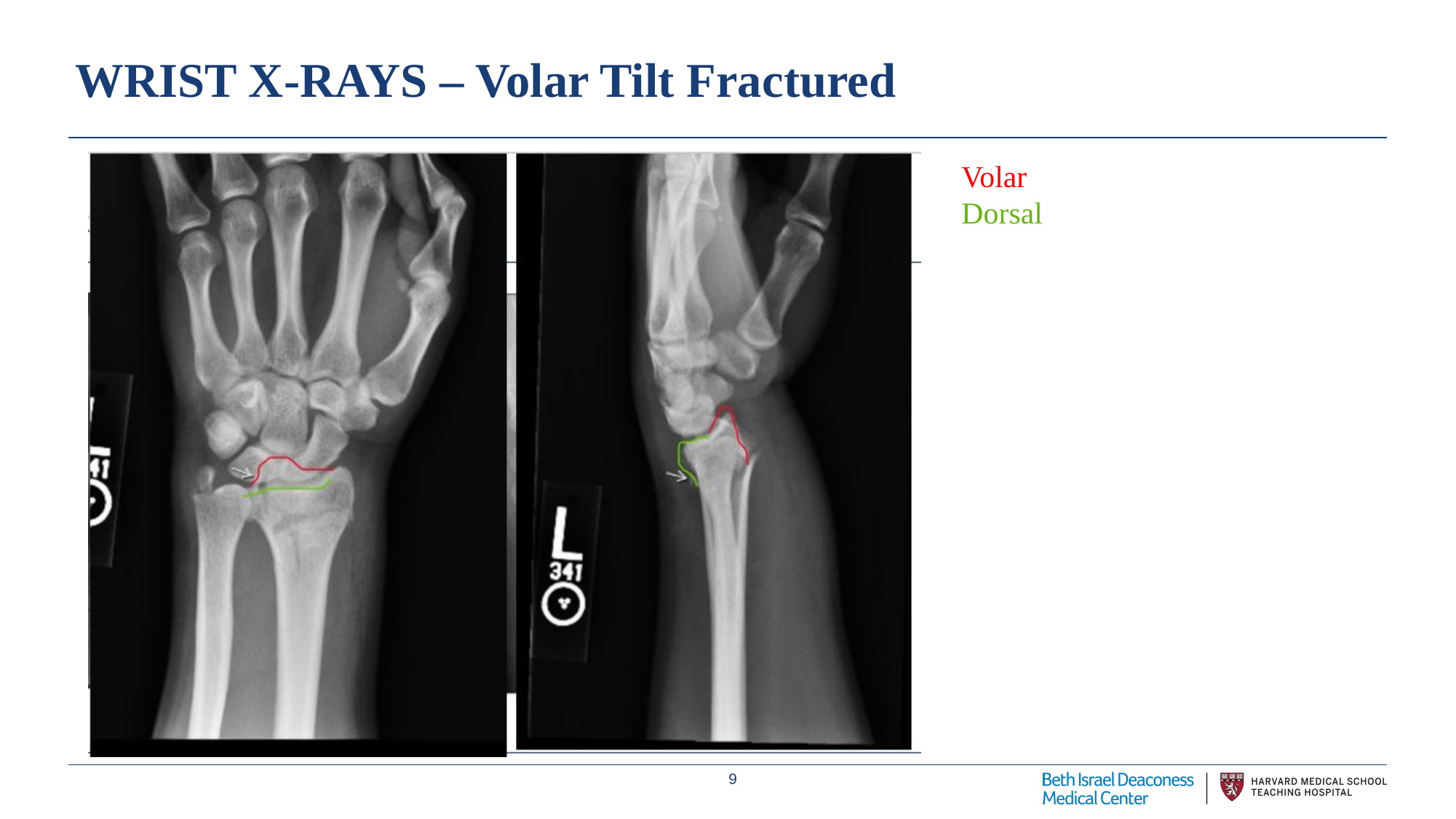

WRIST X-RAYS – Volar Tilt Fractured
Volar
Dorsal

## Slide 10
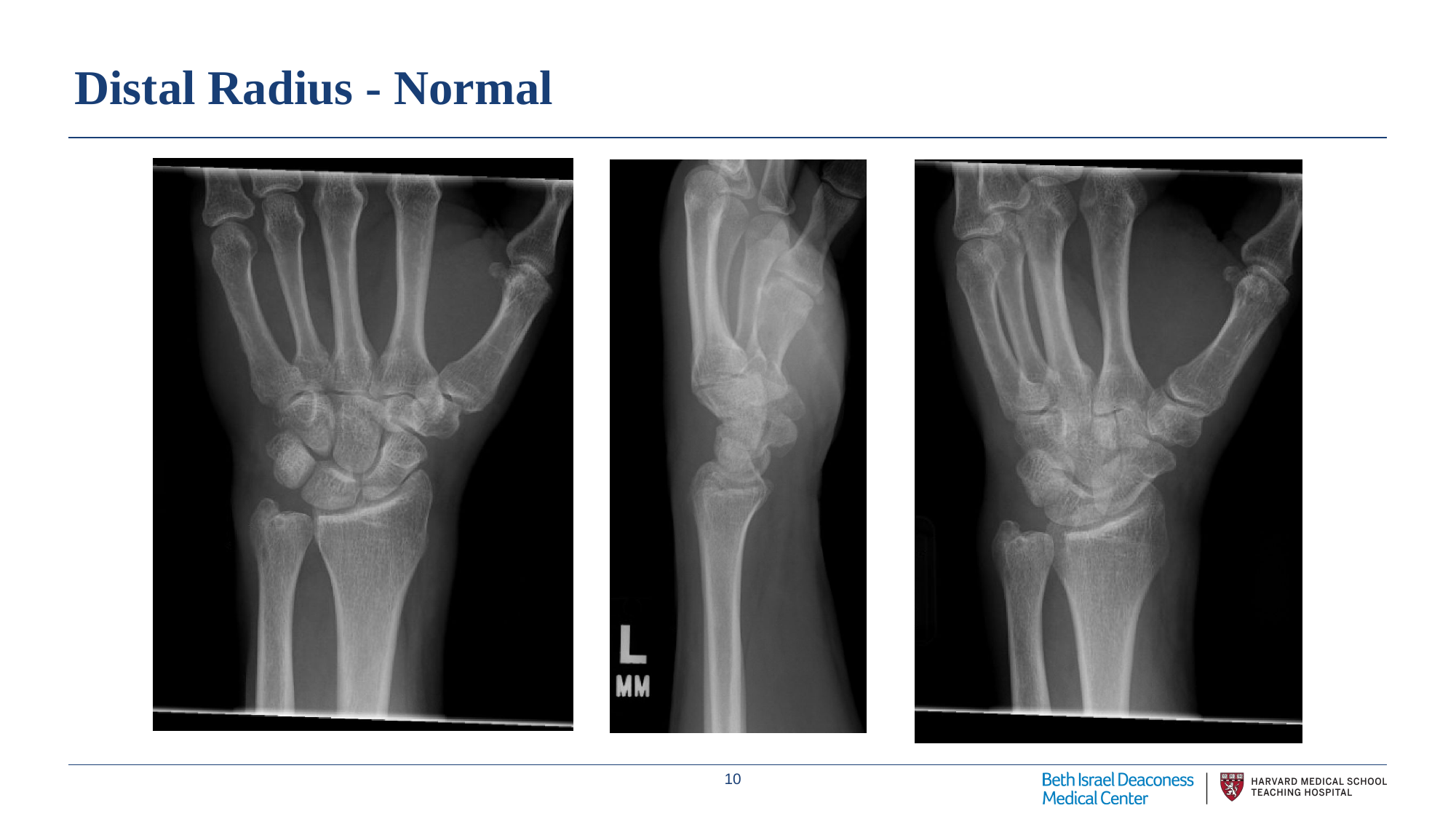

Distal Radius - Normal

## Slide 11
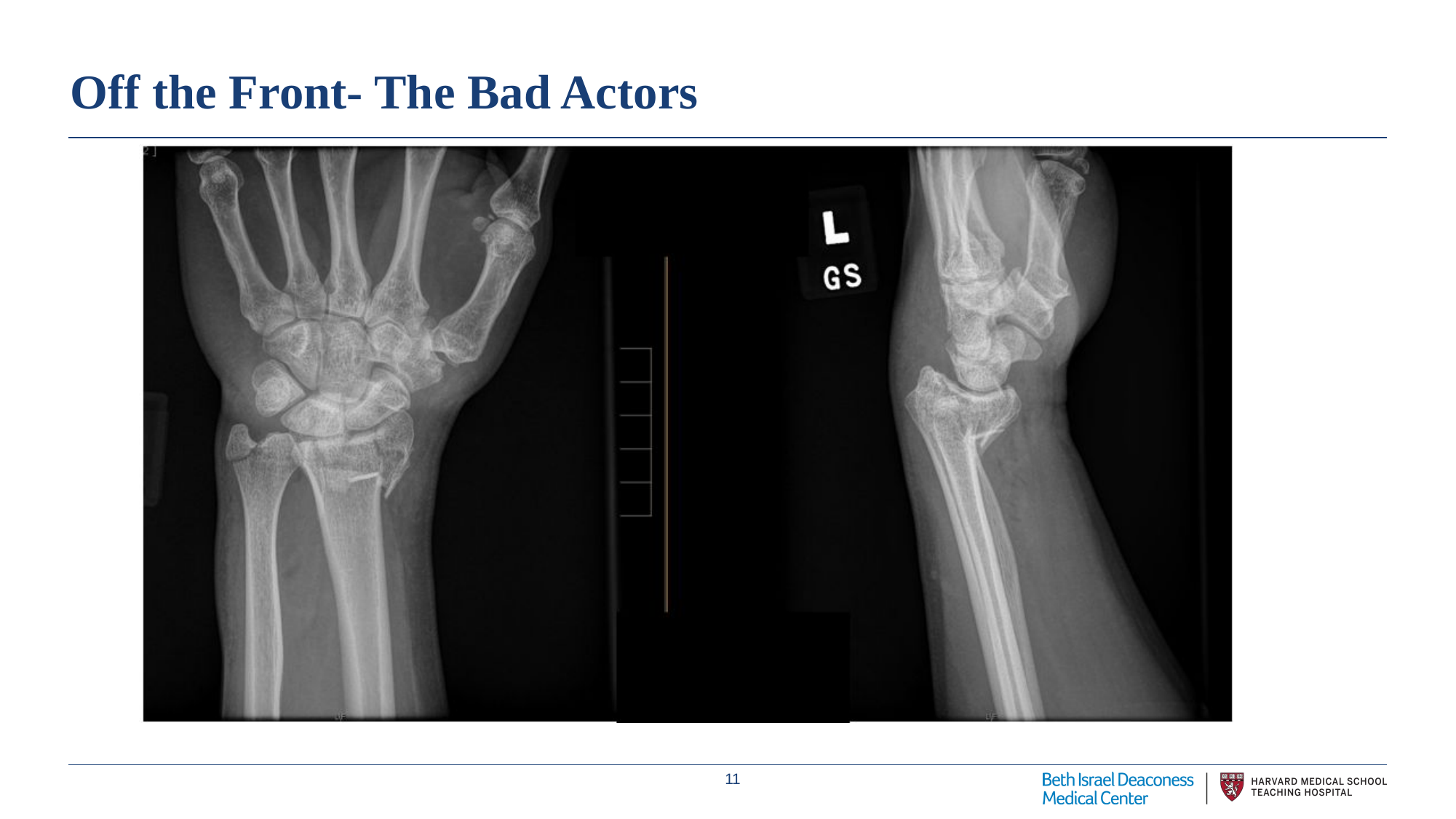

# Off the Front- The Bad Actors

## Slide 12
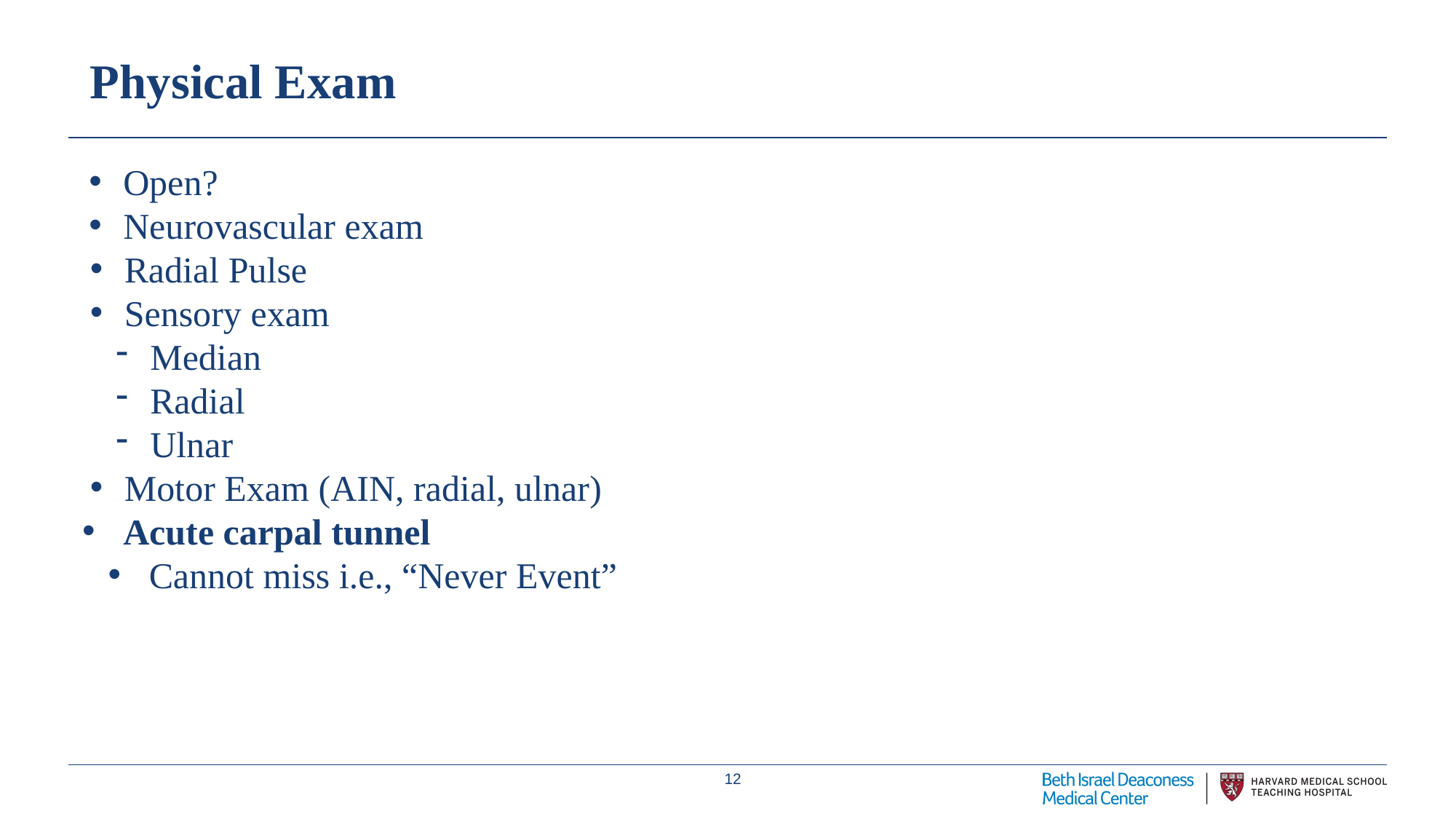

# Physical Exam
Open?
Neurovascular exam
Radial Pulse
Sensory exam
Median
Radial
Ulnar
Motor Exam (AIN, radial, ulnar)
Acute carpal tunnel
Cannot miss i.e., “Never Event”

## Slide 13
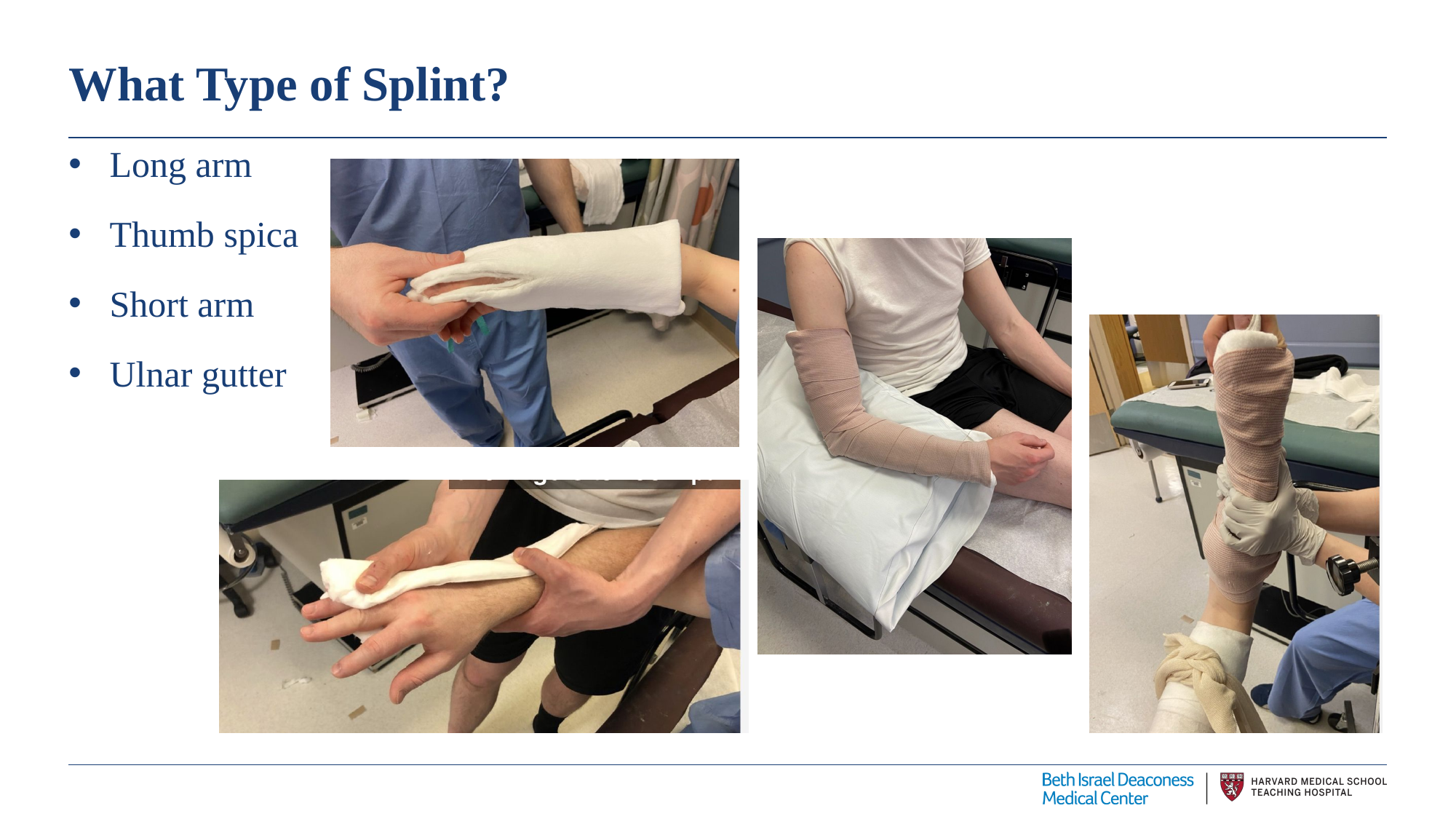

# What Type of Splint?
Long arm
Thumb spica
Short arm
Ulnar gutter

## Slide 14
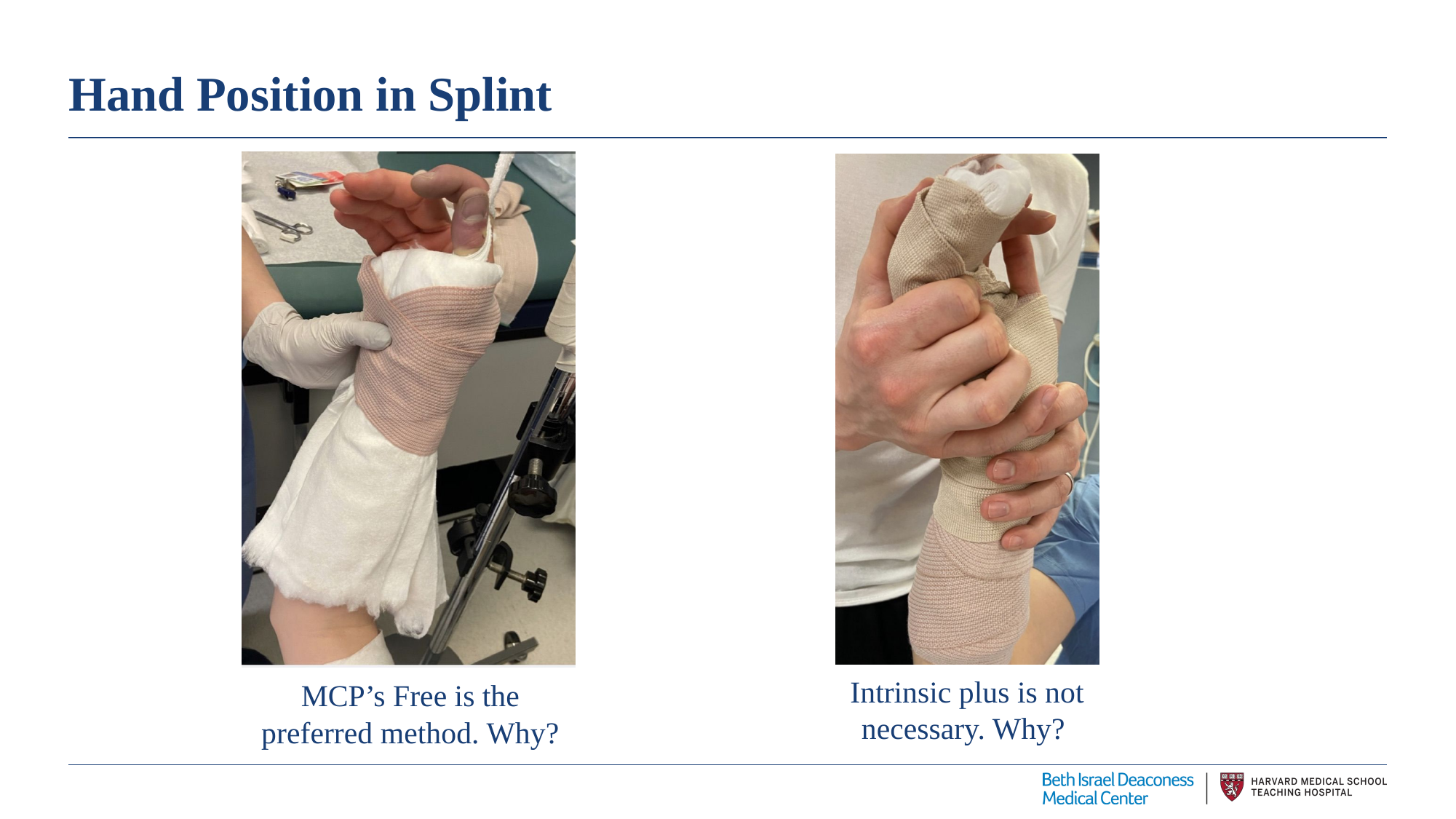

# Hand Position in Splint
Intrinsic plus is not necessary. Why?
MCP’s Free is the preferred method. Why?

## Slide 15
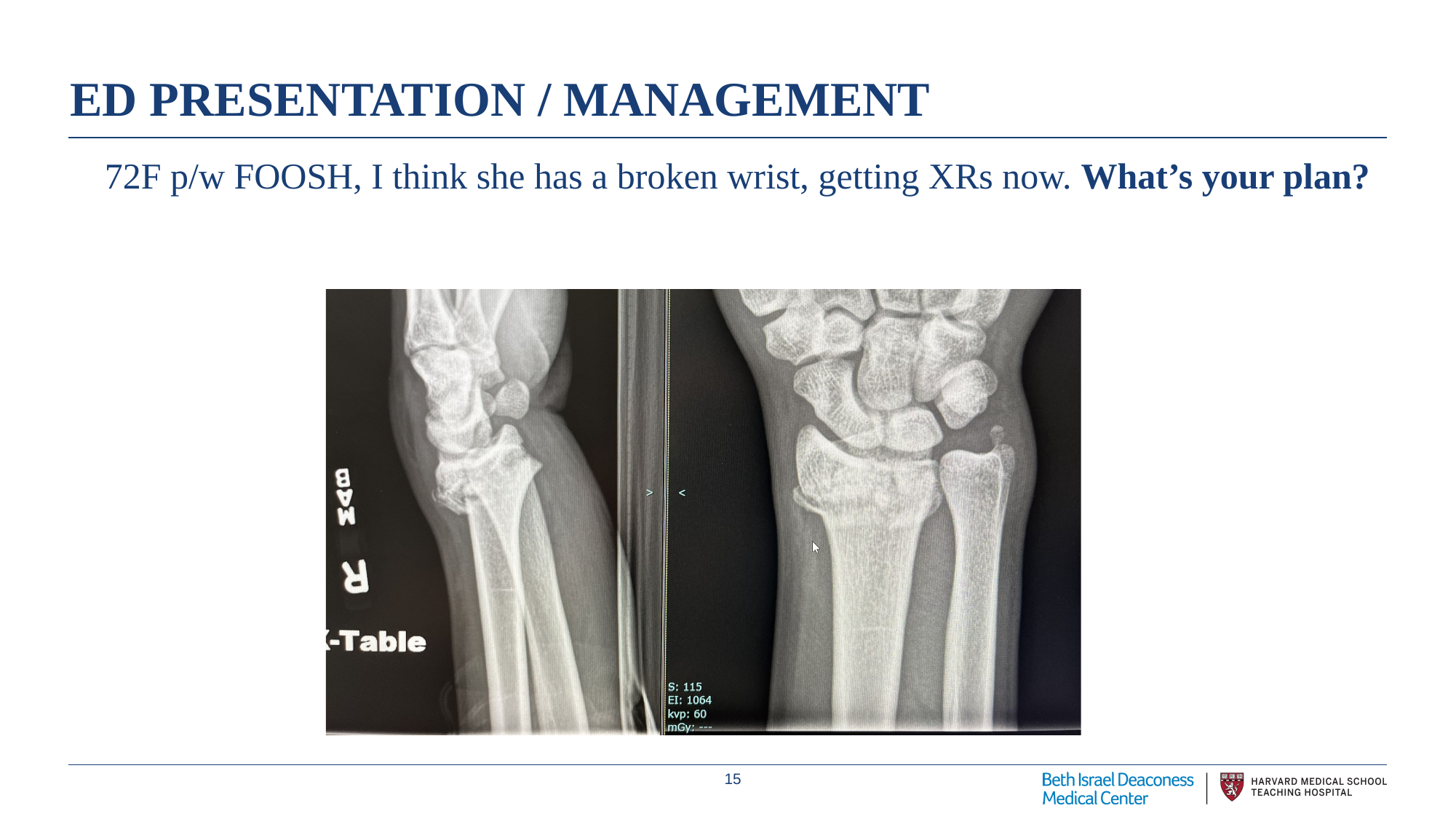

# ED PRESENTATION / MANAGEMENT
72F p/w FOOSH, I think she has a broken wrist, getting XRs now. What’s your plan?

## Slide 16
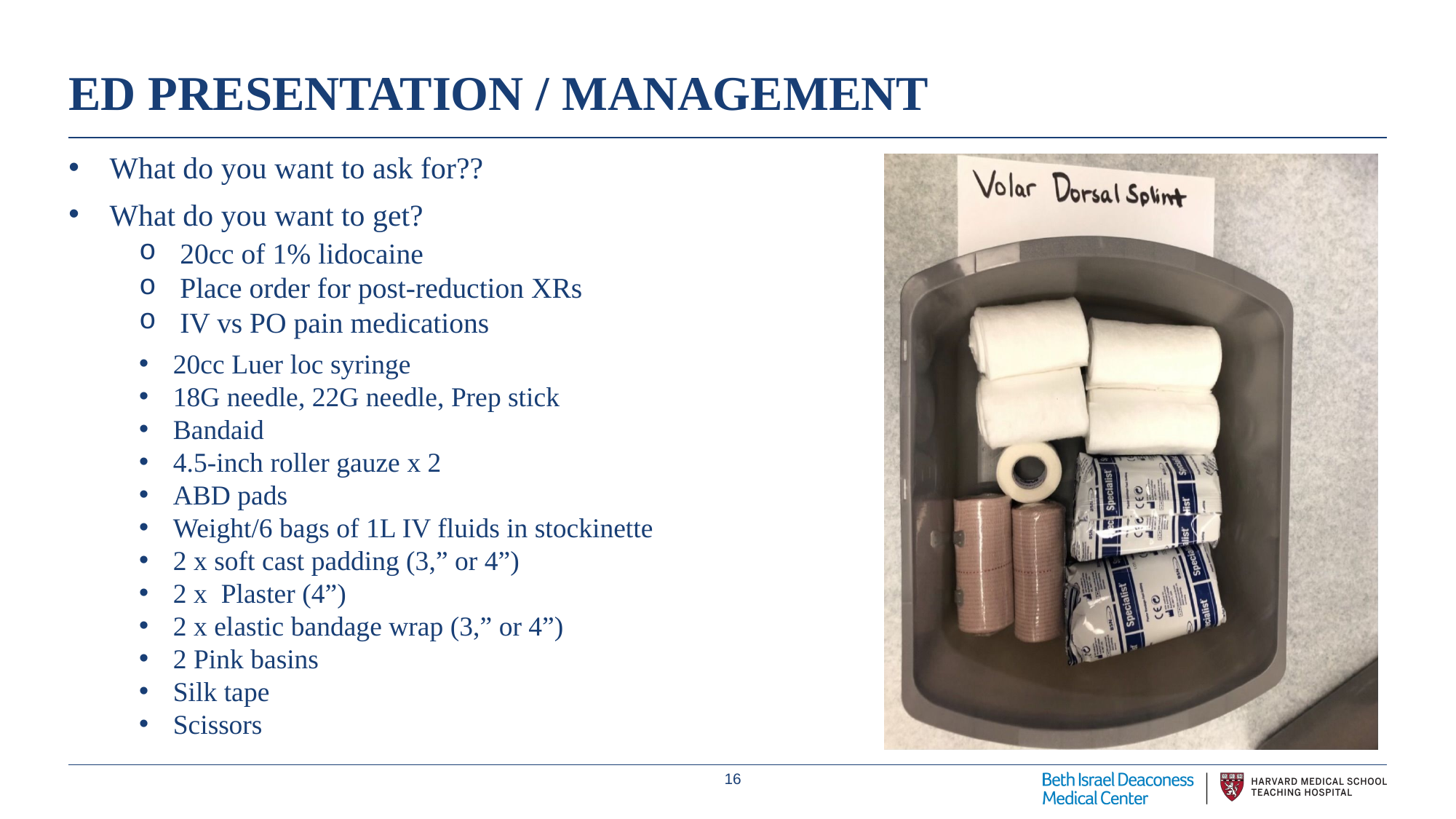

# ED PRESENTATION / MANAGEMENT
What do you want to ask for??
What do you want to get?
20cc of 1% lidocaine
Place order for post-reduction XRs
IV vs PO pain medications
20cc Luer loc syringe
18G needle, 22G needle, Prep stick
Bandaid
4.5-inch roller gauze x 2
ABD pads
Weight/6 bags of 1L IV fluids in stockinette
2 x soft cast padding (3,” or 4”)
2 x Plaster (4”)
2 x elastic bandage wrap (3,” or 4”)
2 Pink basins
Silk tape
Scissors

## Slide 17
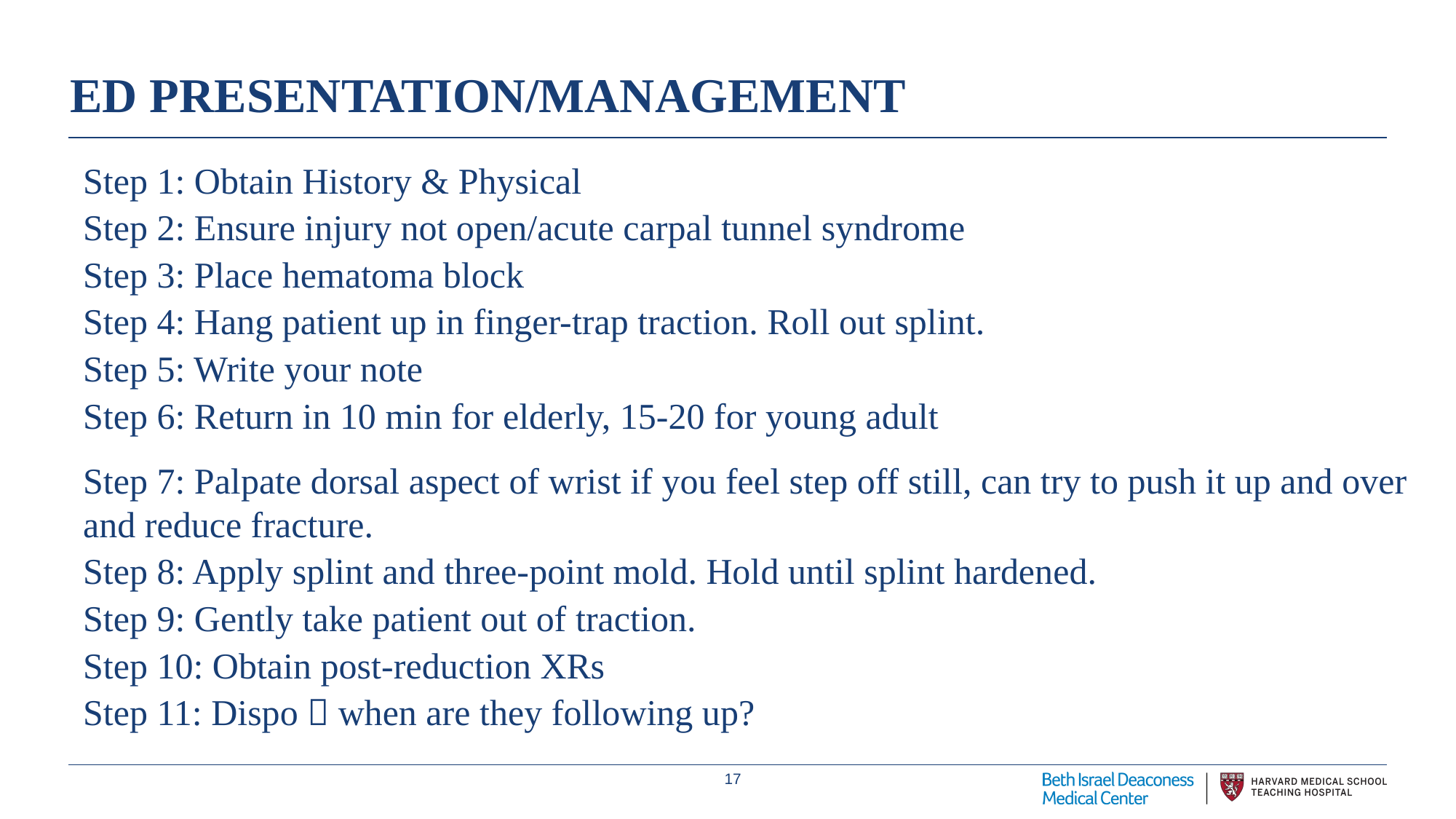

# ED PRESENTATION/MANAGEMENT
Step 1: Obtain History & Physical
Step 2: Ensure injury not open/acute carpal tunnel syndrome
Step 3: Place hematoma block
Step 4: Hang patient up in finger-trap traction. Roll out splint.
Step 5: Write your note
Step 6: Return in 10 min for elderly, 15-20 for young adult
Step 7: Palpate dorsal aspect of wrist if you feel step off still, can try to push it up and over and reduce fracture.
Step 8: Apply splint and three-point mold. Hold until splint hardened.
Step 9: Gently take patient out of traction.
Step 10: Obtain post-reduction XRs
Step 11: Dispo  when are they following up?

## Slide 18
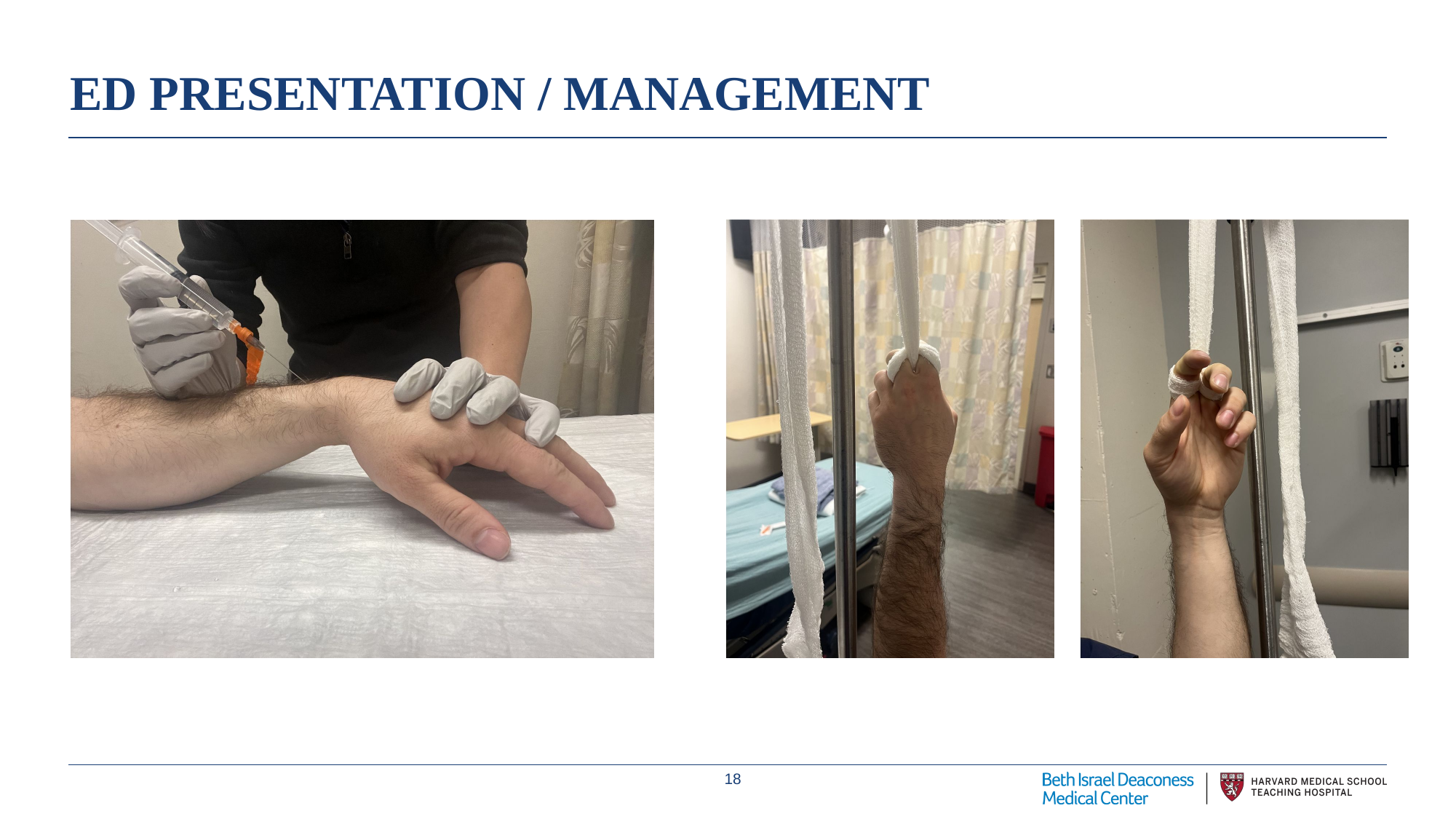

# ED PRESENTATION / MANAGEMENT

## Slide 19
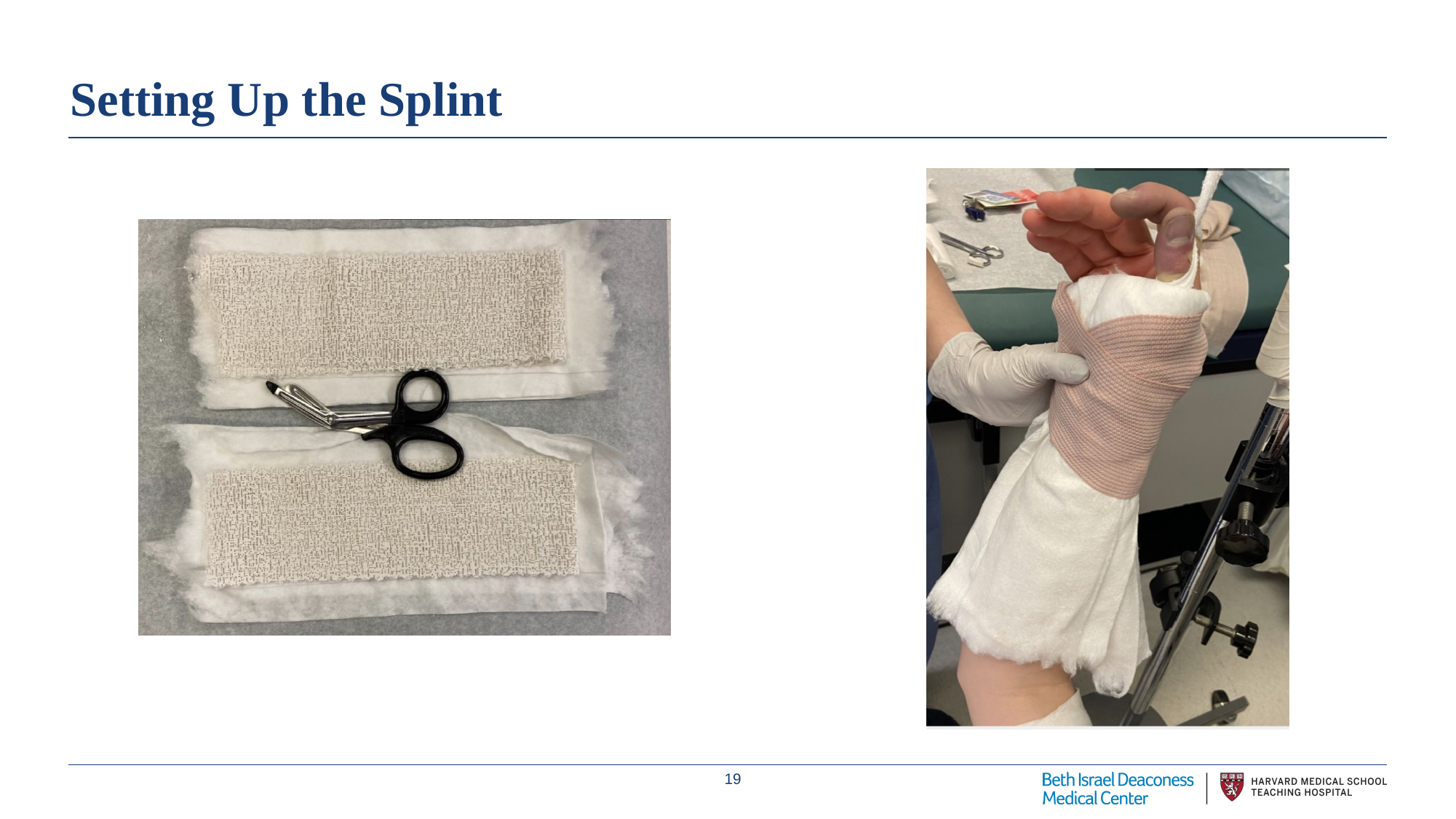

# Setting Up the Splint

## Slide 20
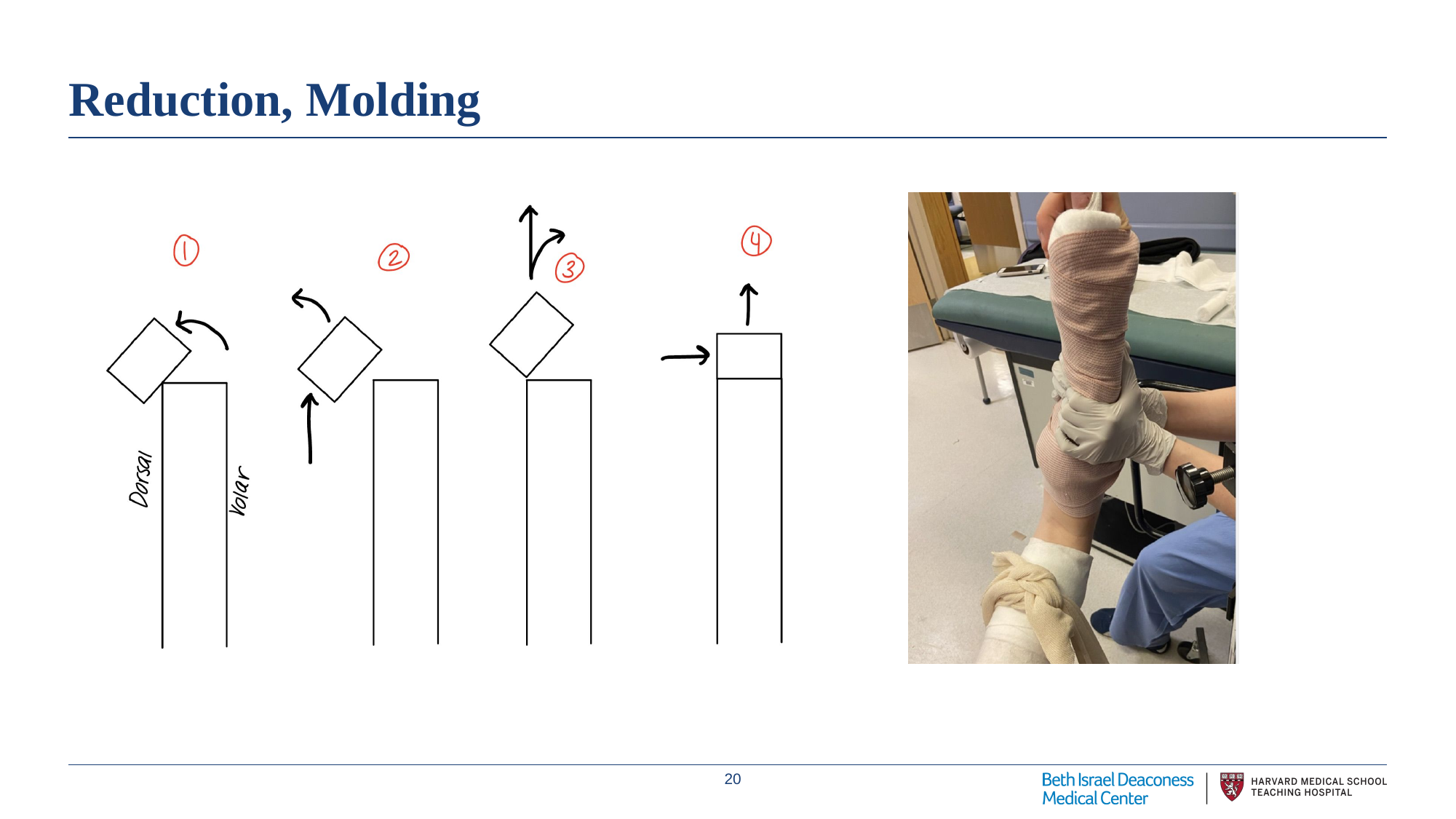

Reduction, Molding

## Slide 21
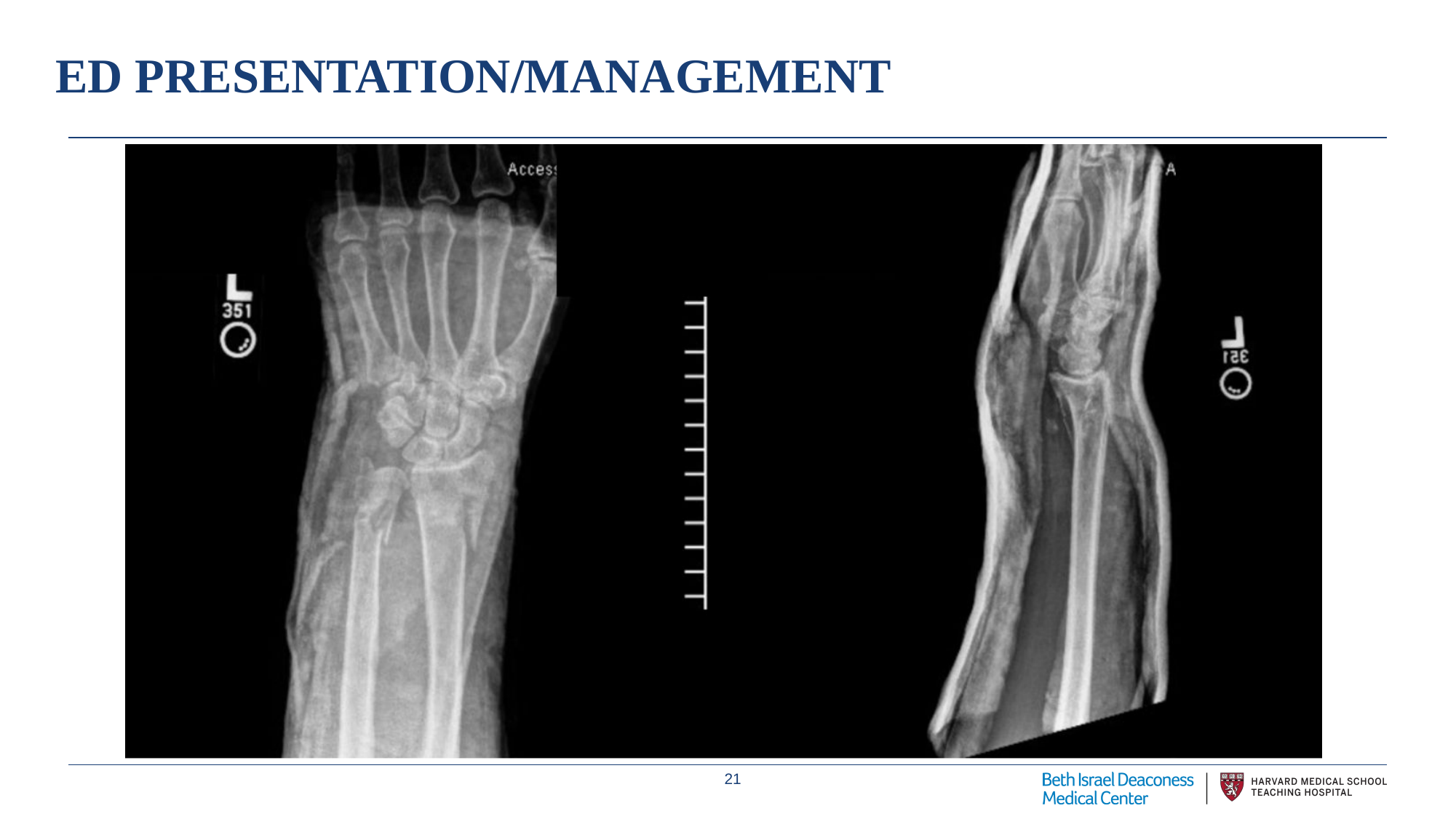

# ED PRESENTATION/MANAGEMENT

## Slide 22
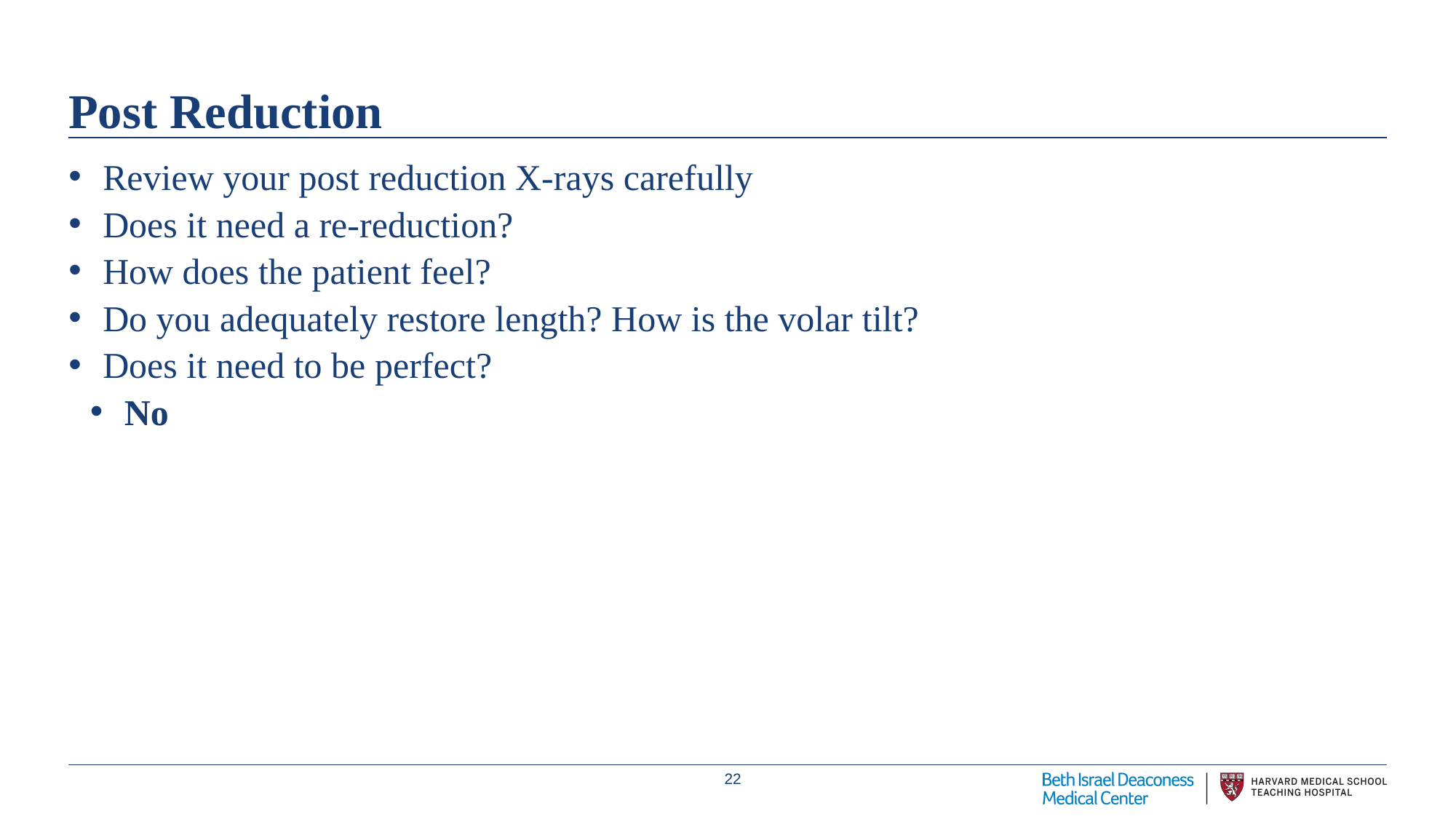

# Post Reduction
Review your post reduction X-rays carefully
Does it need a re-reduction?
How does the patient feel?
Do you adequately restore length? How is the volar tilt?
Does it need to be perfect?
No

## Slide 23
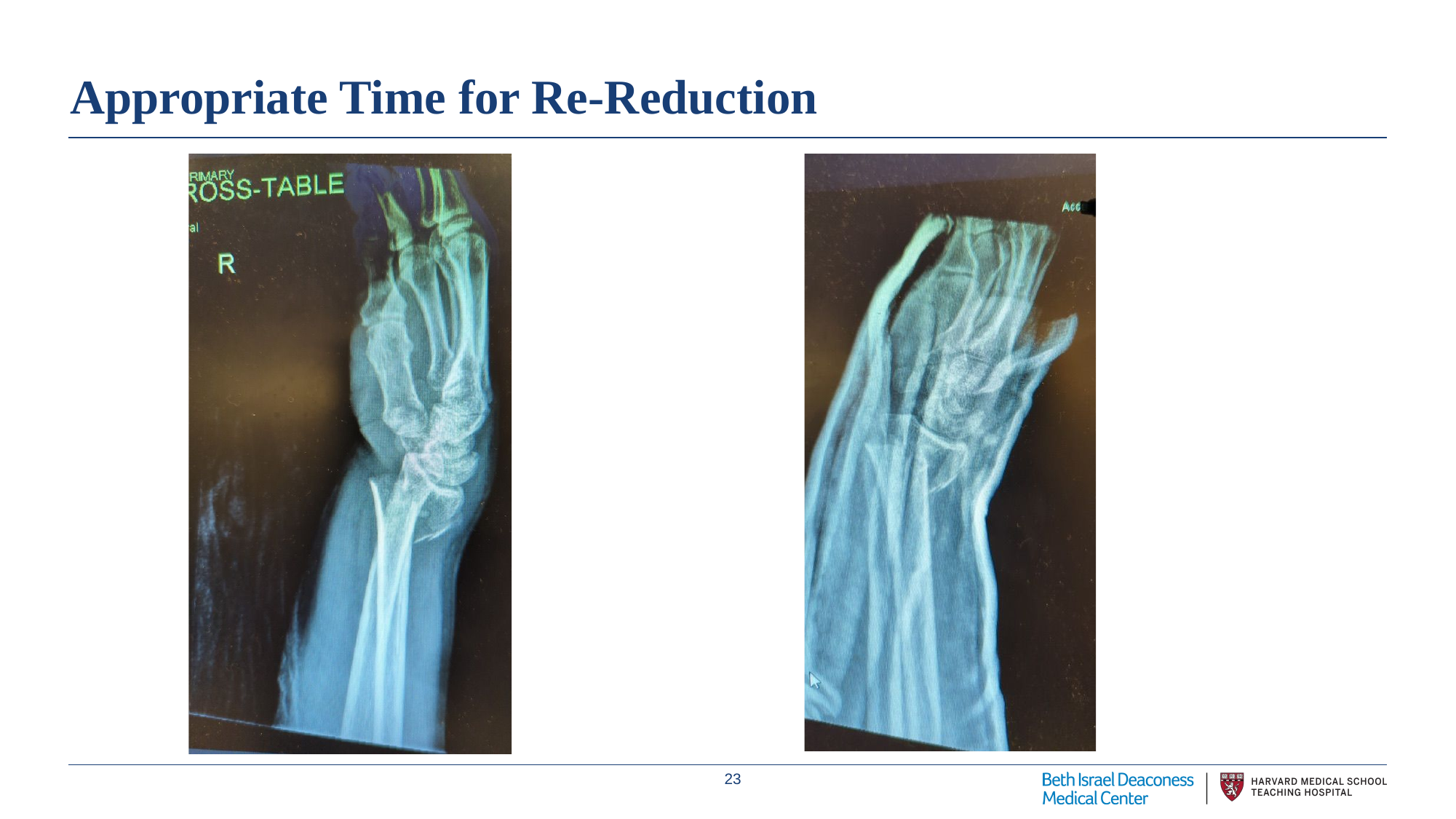

# Appropriate Time for Re-Reduction

## Slide 24
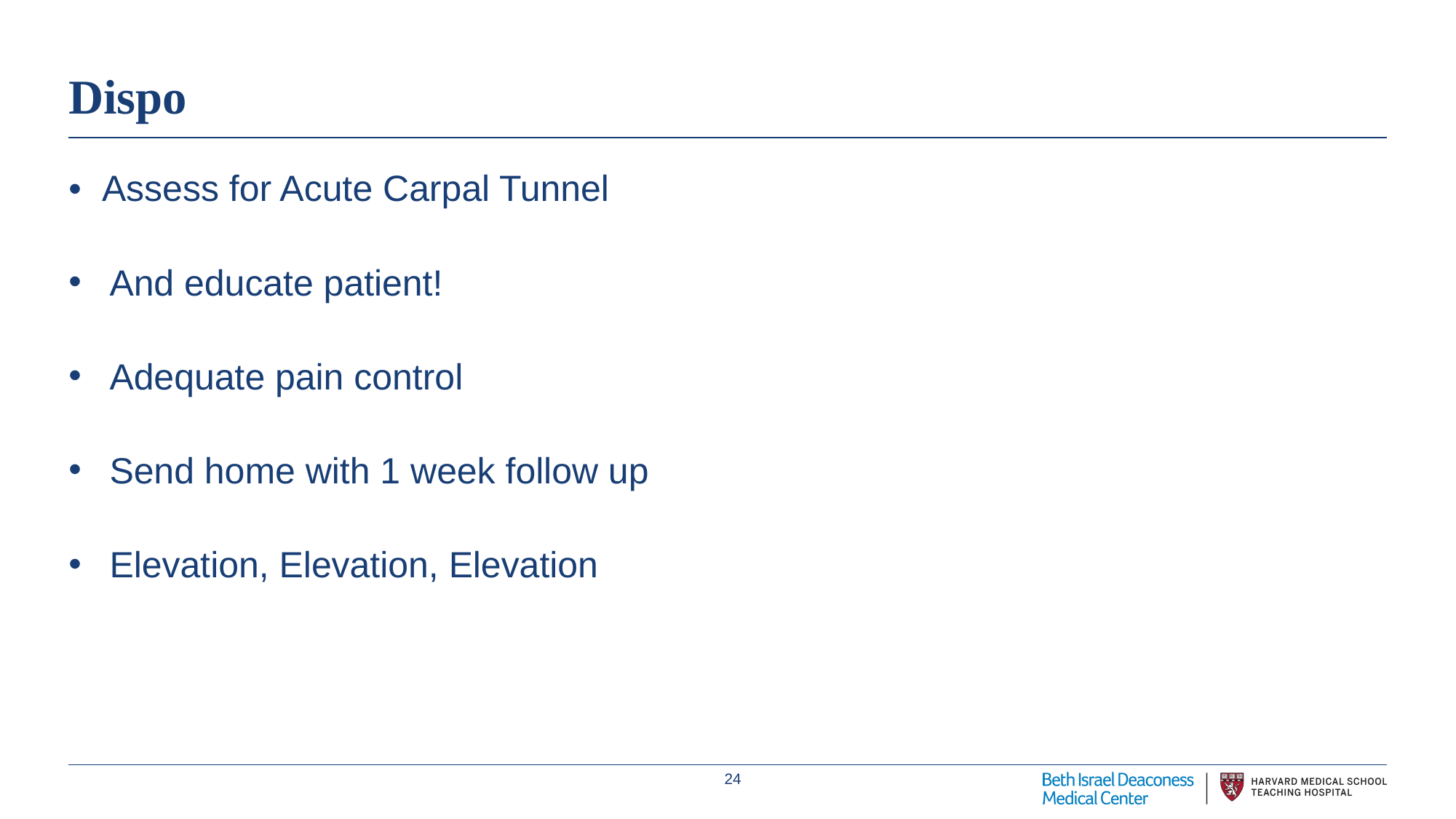

# Dispo
• Assess for Acute Carpal Tunnel
And educate patient!
Adequate pain control
Send home with 1 week follow up
Elevation, Elevation, Elevation

## Slide 25
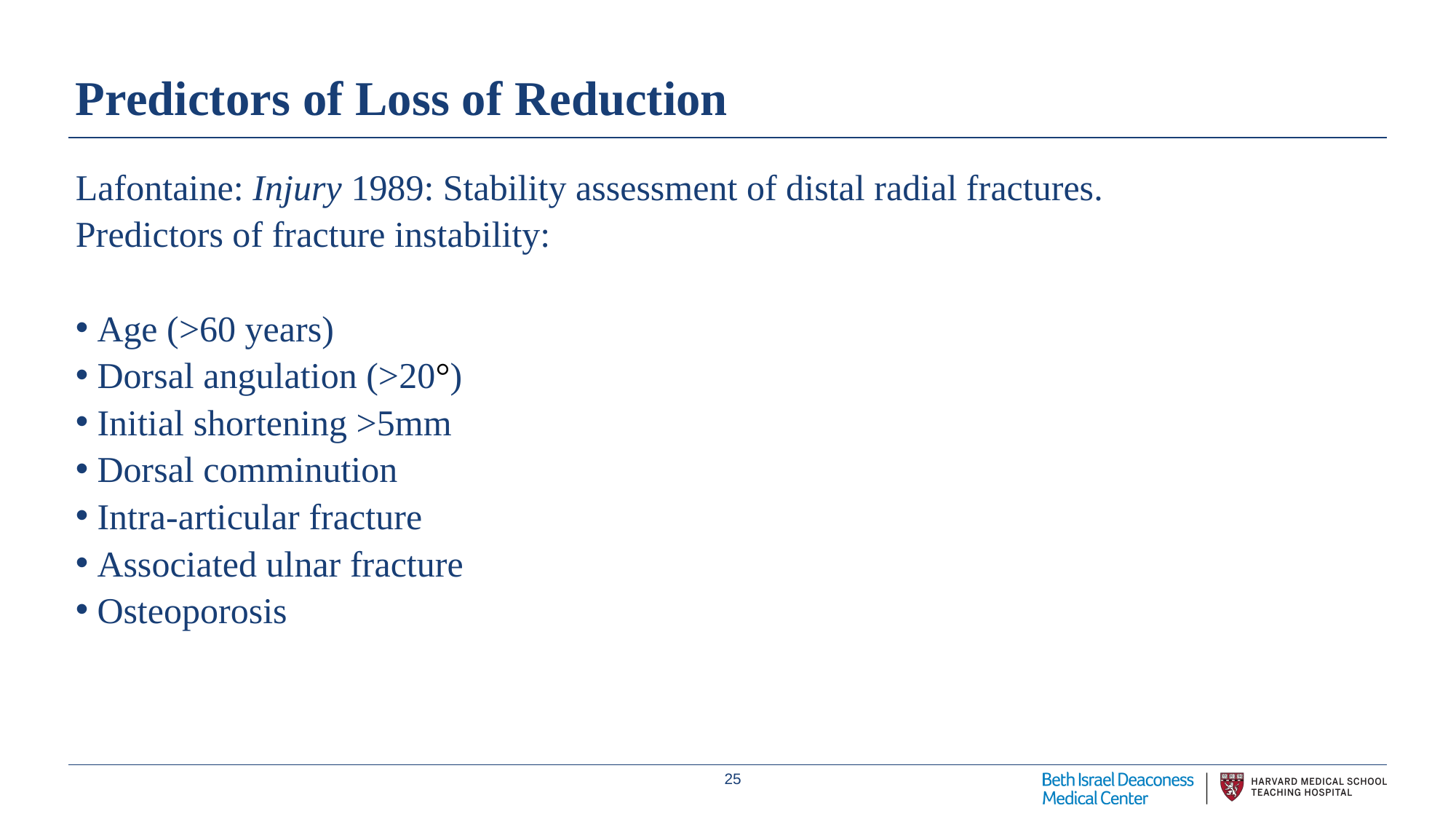

Predictors of Loss of Reduction
Lafontaine: Injury 1989: Stability assessment of distal radial fractures.
Predictors of fracture instability:
Age (>60 years)
Dorsal angulation (>20°)
Initial shortening >5mm
Dorsal comminution
Intra-articular fracture
Associated ulnar fracture
Osteoporosis

## Slide 26
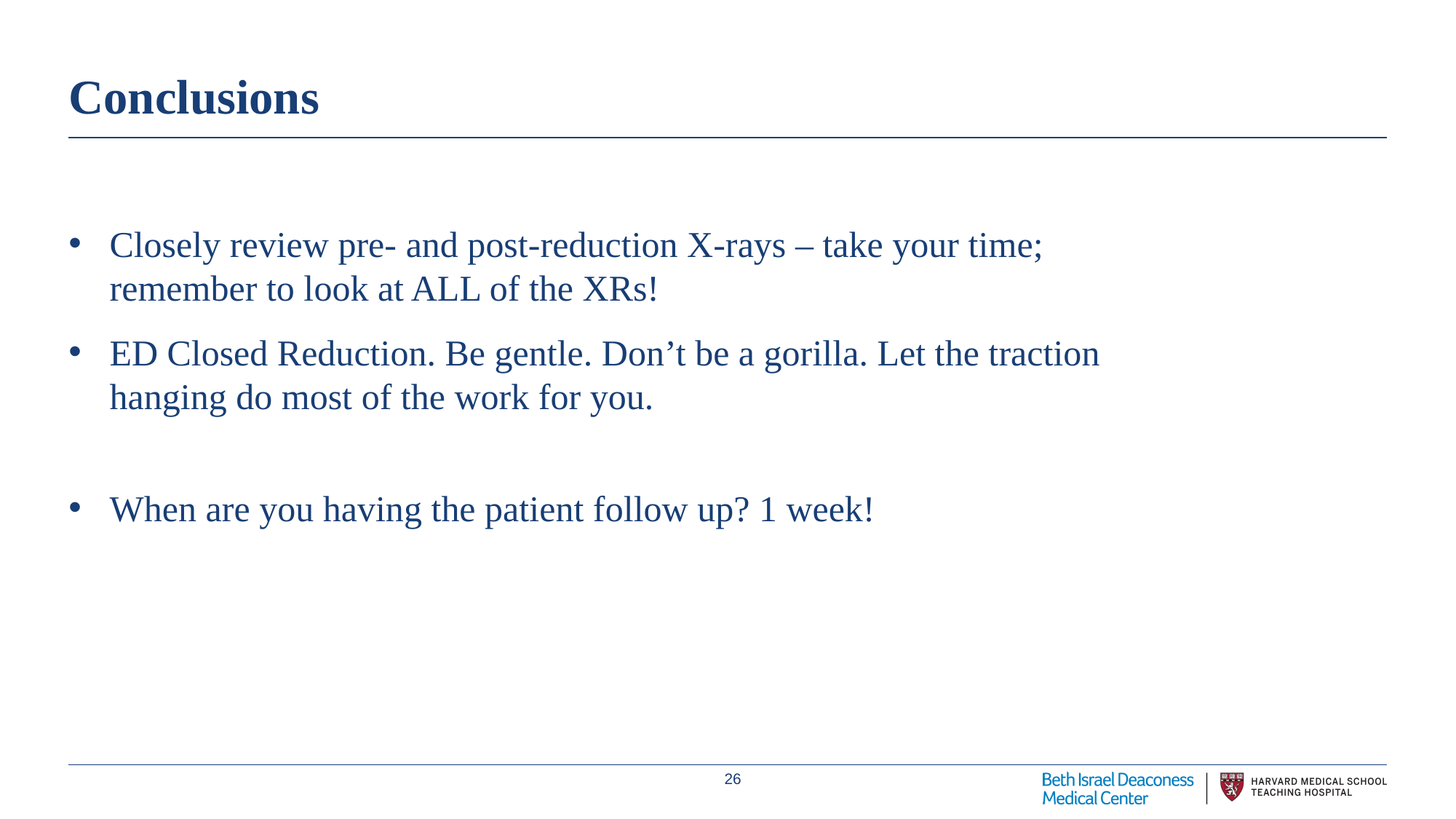

# Conclusions
Closely review pre- and post-reduction X-rays – take your time; remember to look at ALL of the XRs!
ED Closed Reduction. Be gentle. Don’t be a gorilla. Let the traction hanging do most of the work for you.
When are you having the patient follow up? 1 week!

## Slide 27
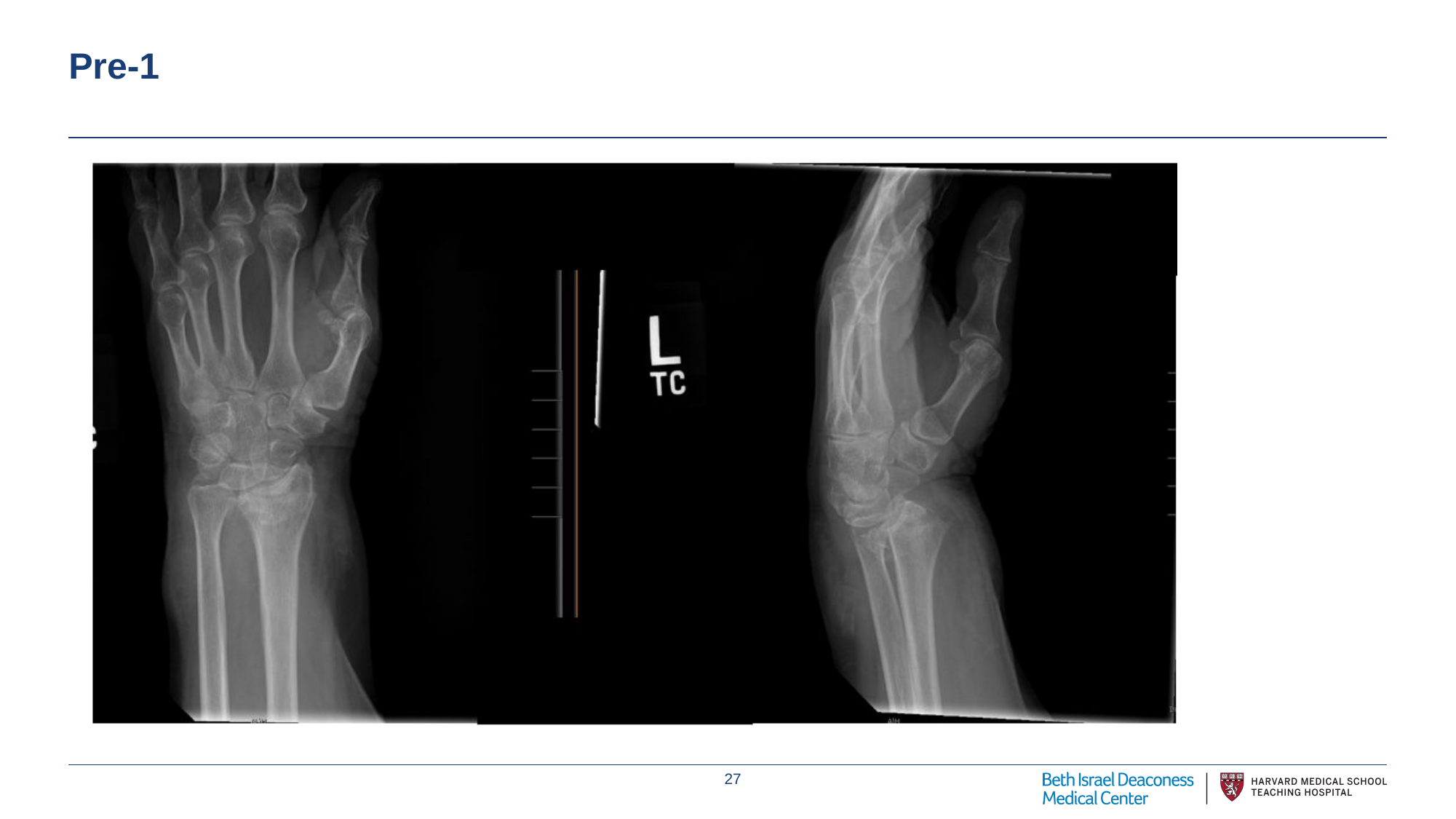

# Pre-1

## Slide 28
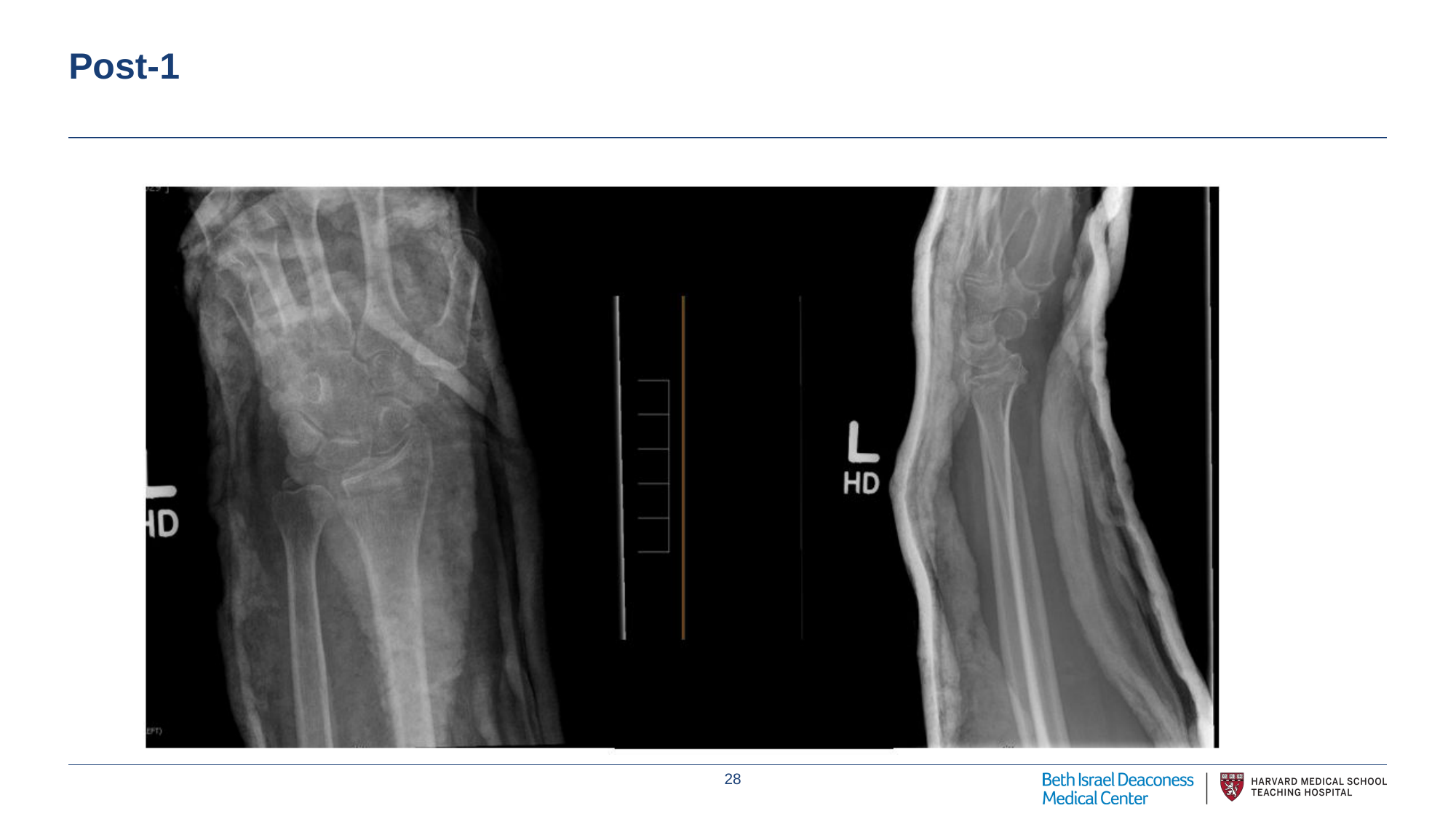

# Post-1

## Slide 29
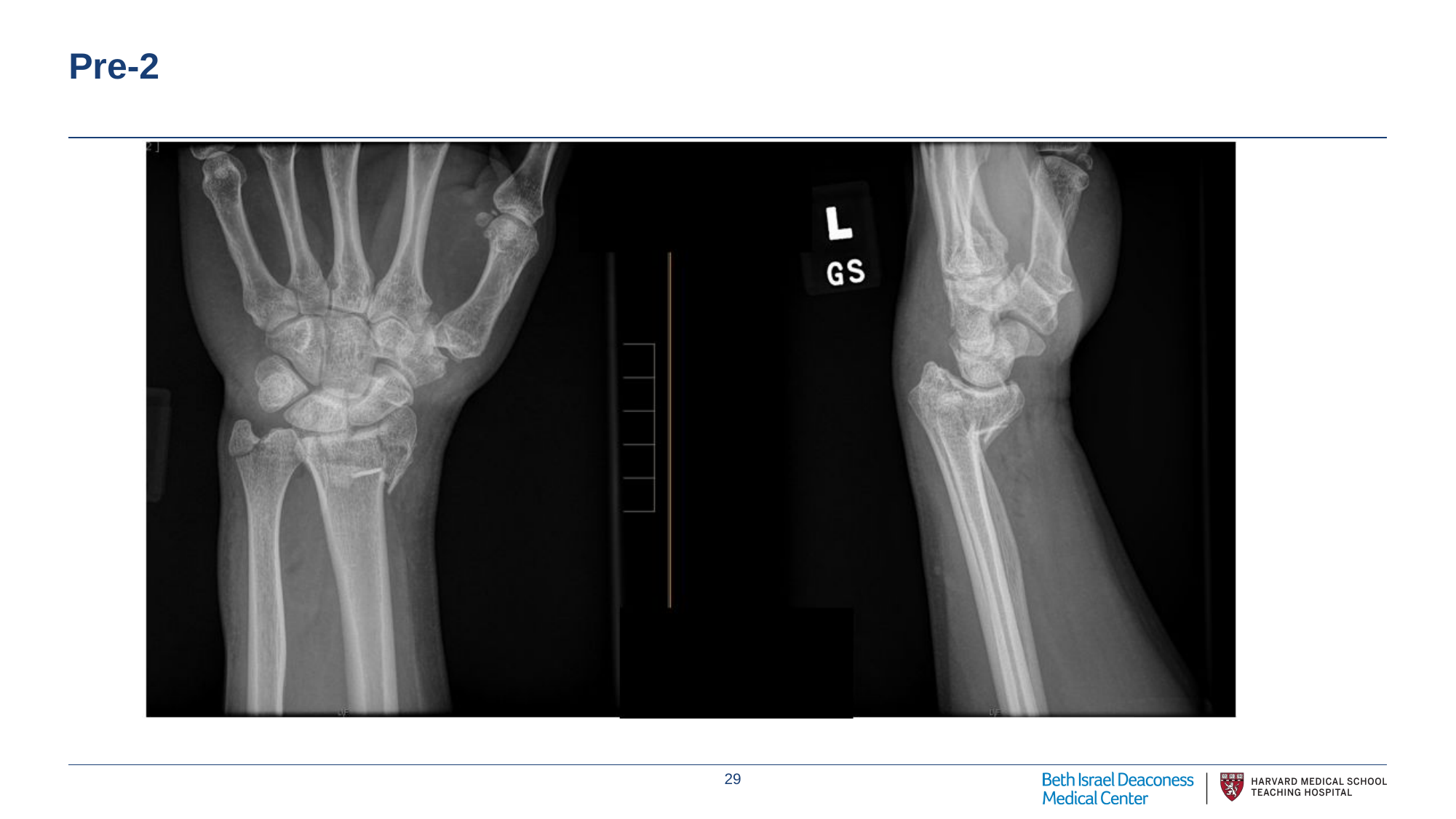

# Pre-2

## Slide 30
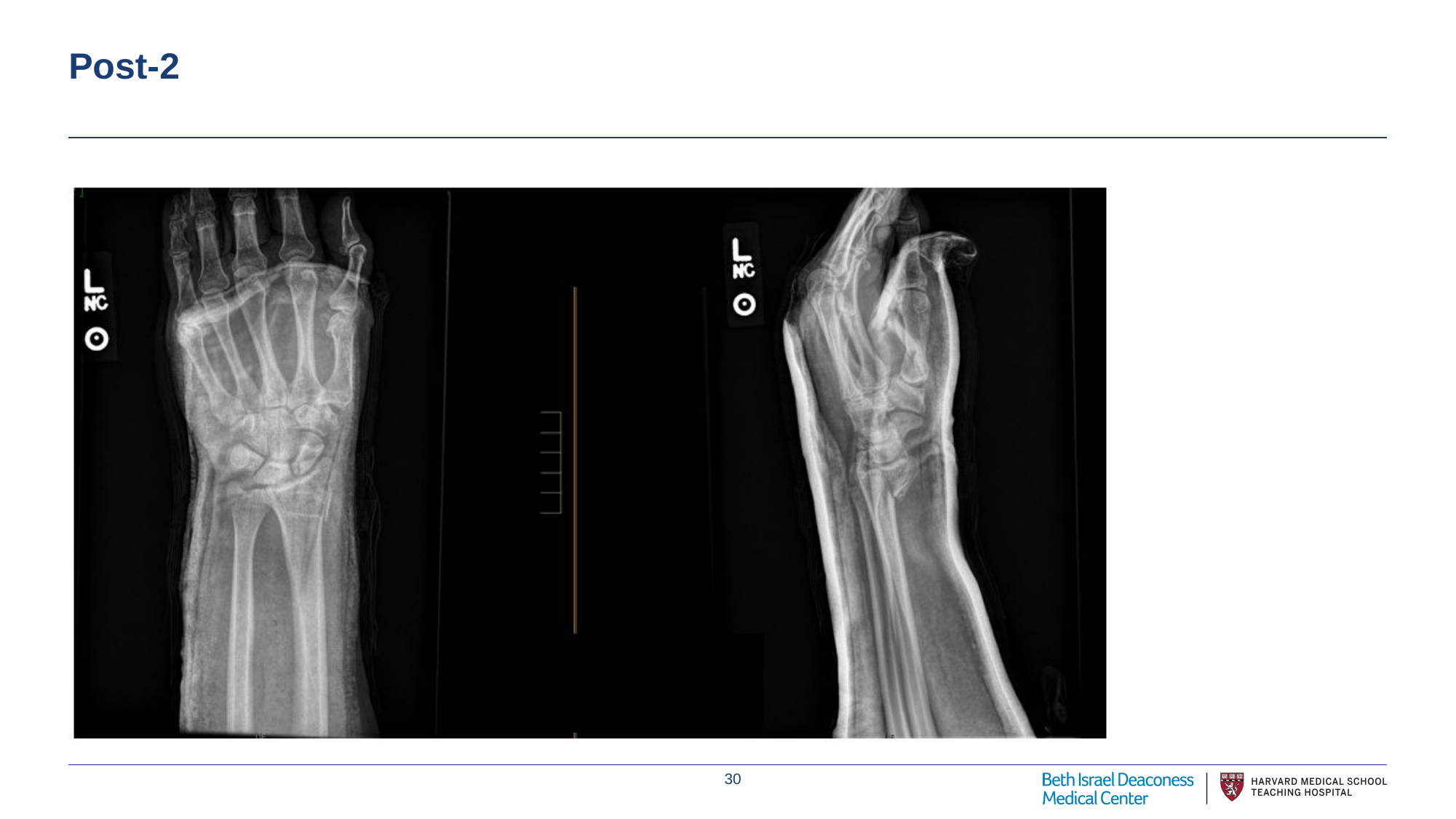

# Post-2

## Slide 31
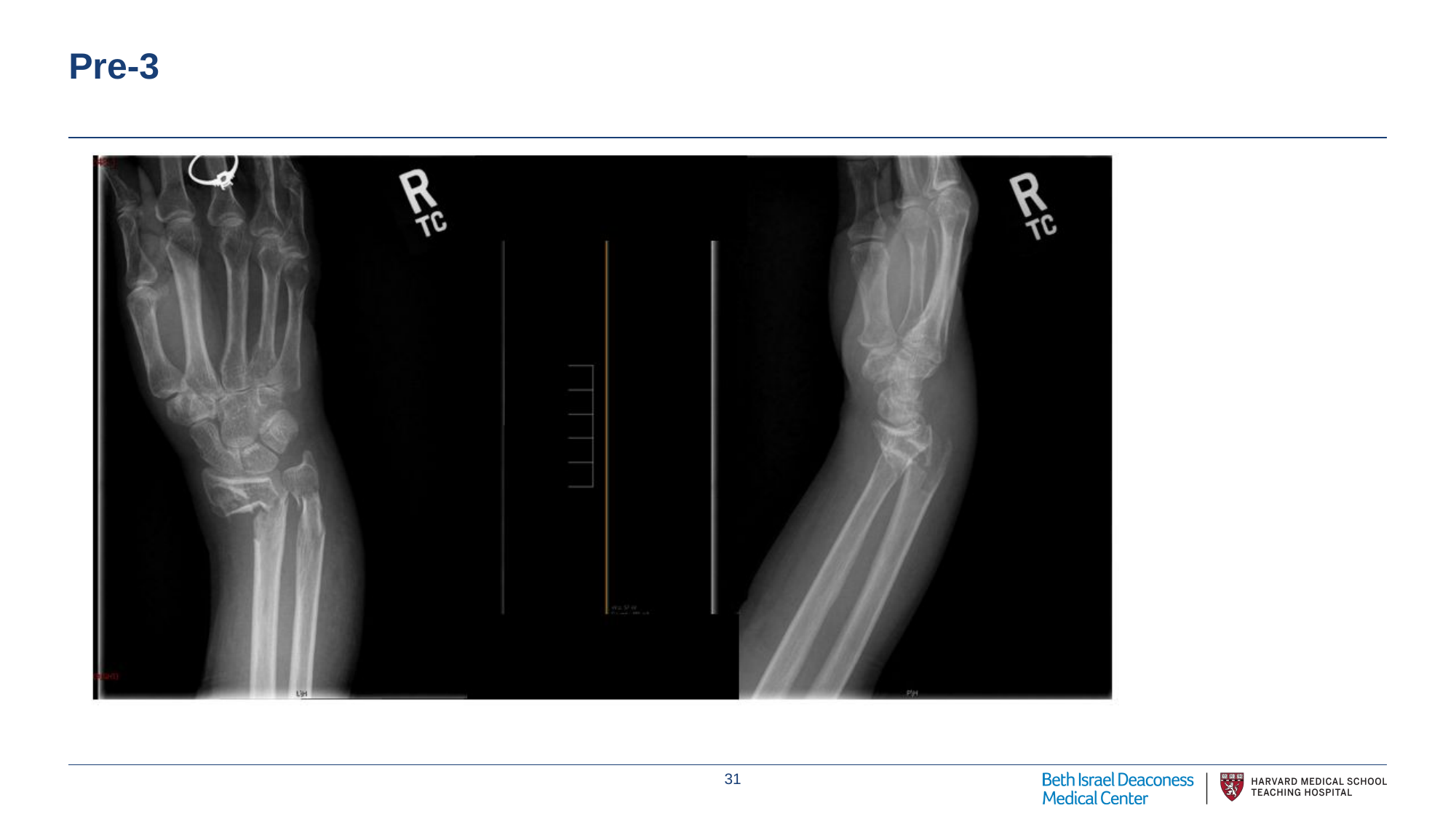

# Pre-3

## Slide 32
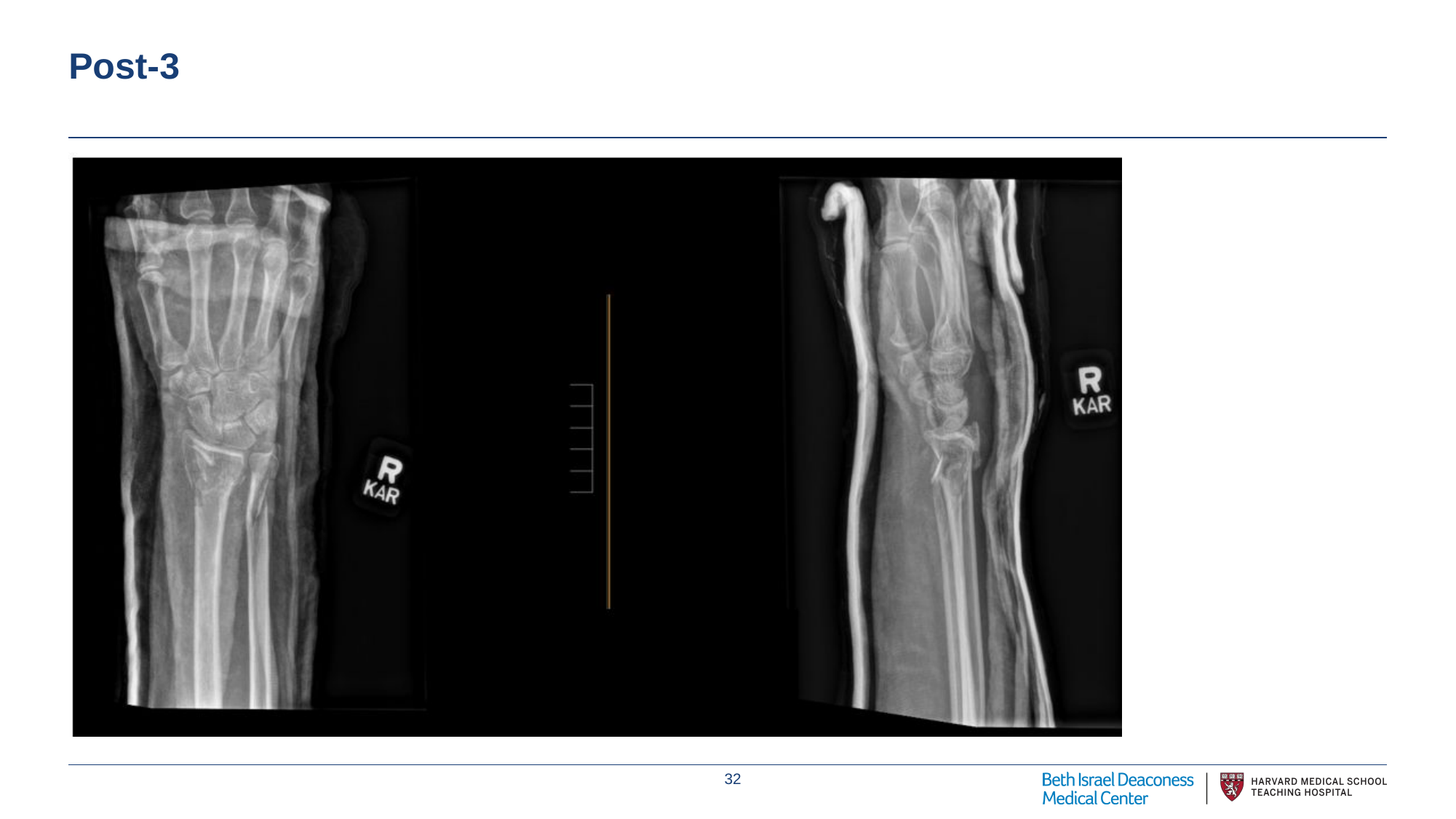

# Post-3

## Slide 33
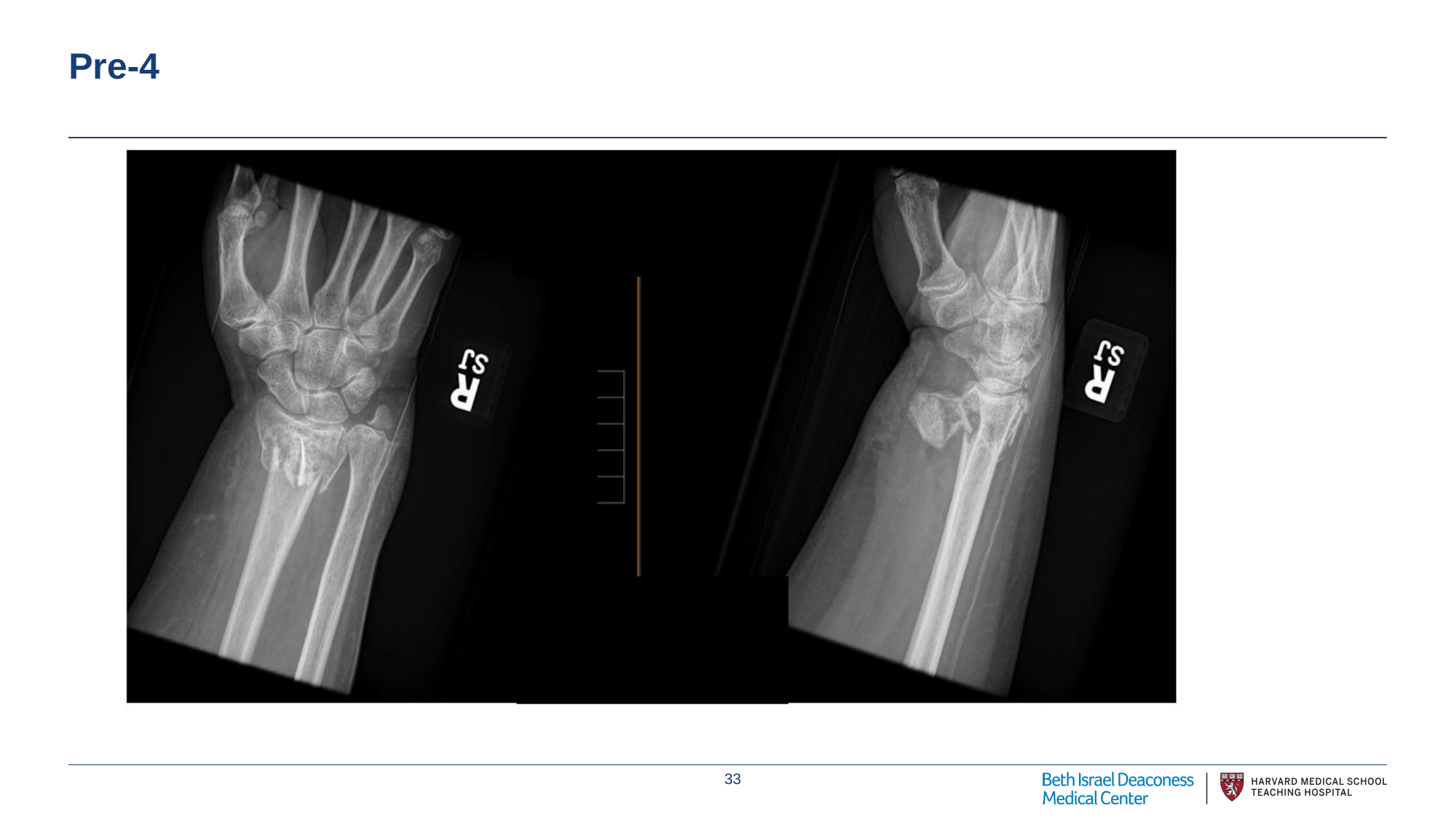

# Pre-4

## Slide 34
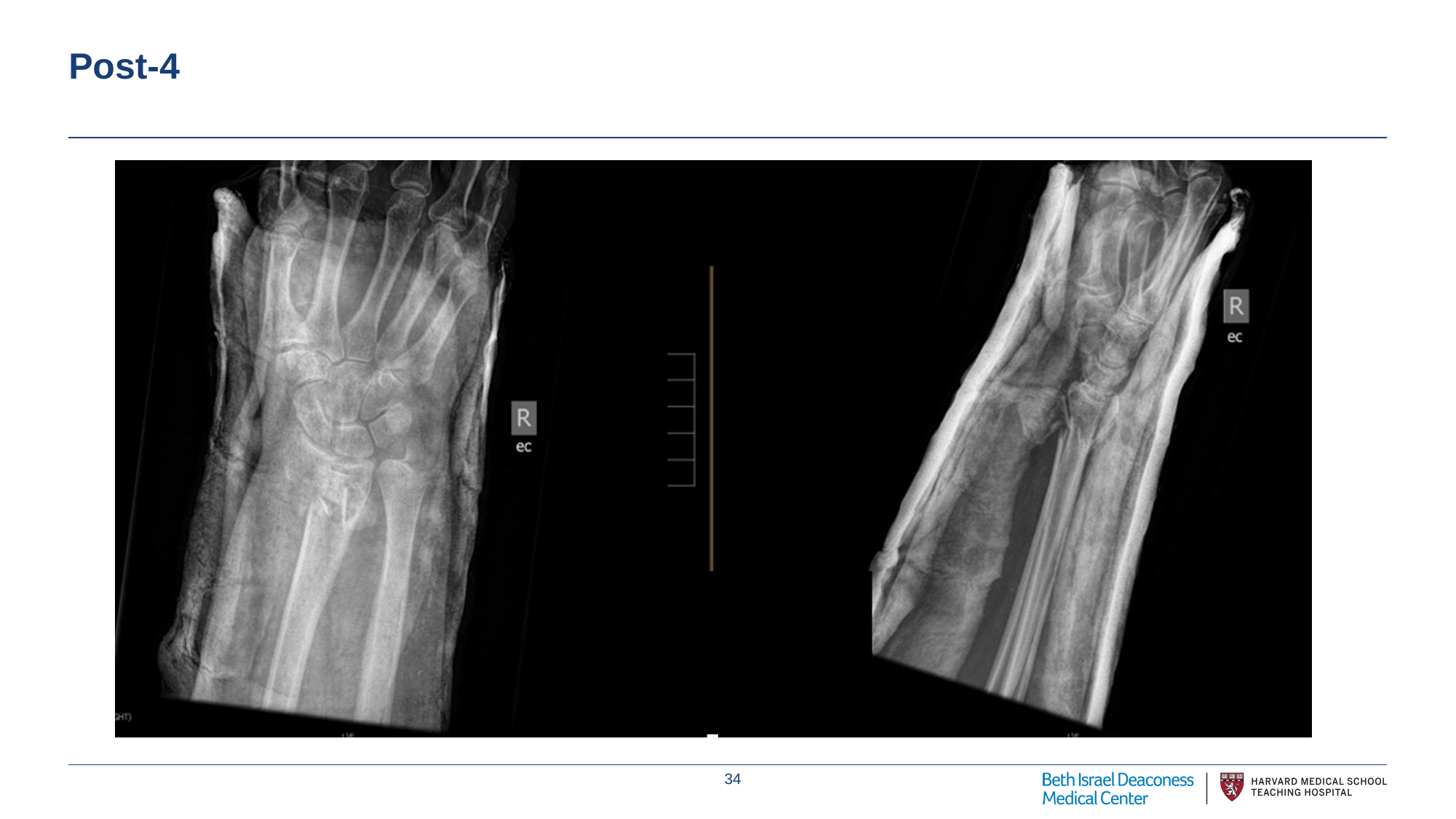

# Post-4

## Slide 35
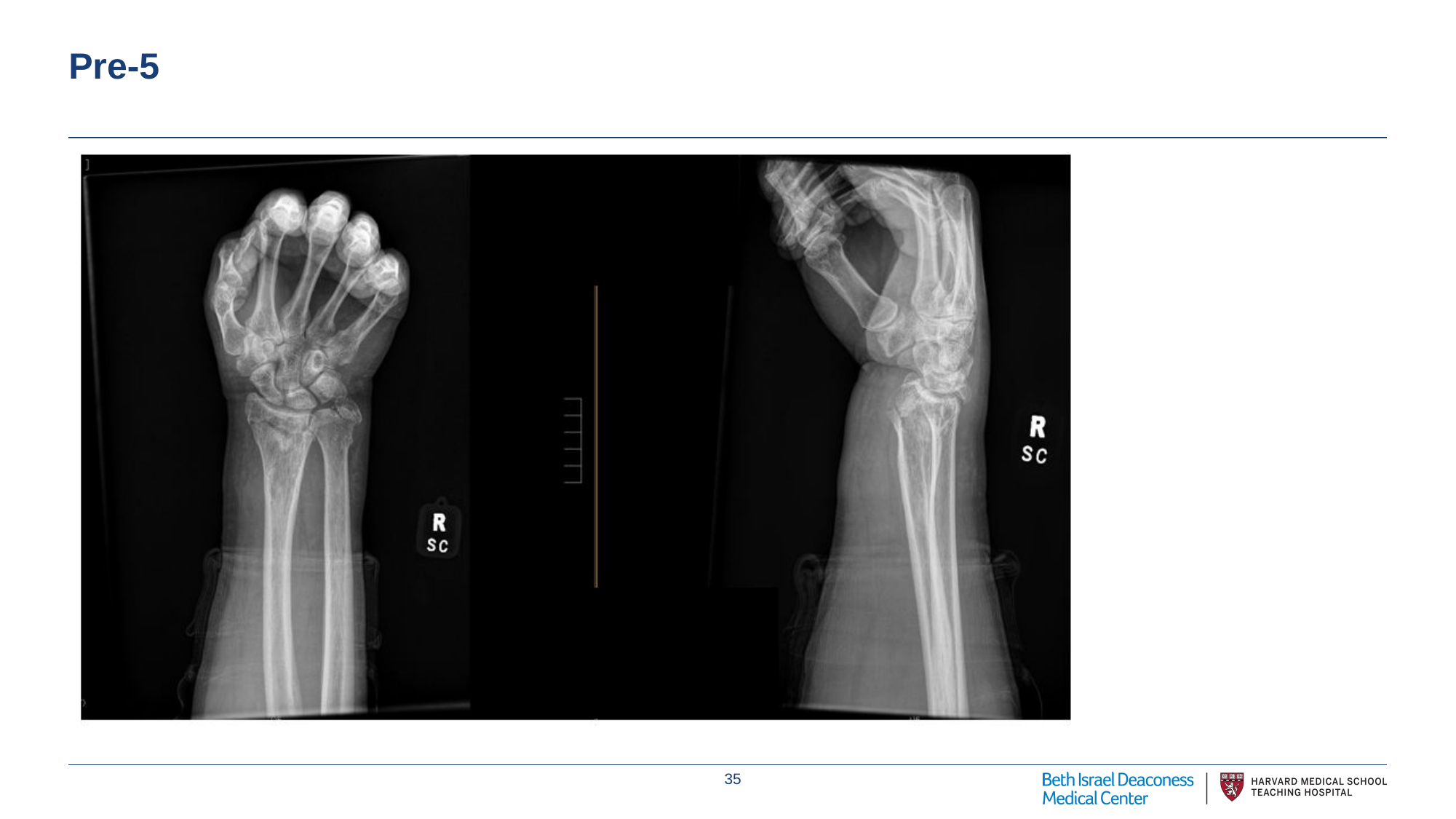

# Pre-5

## Slide 36
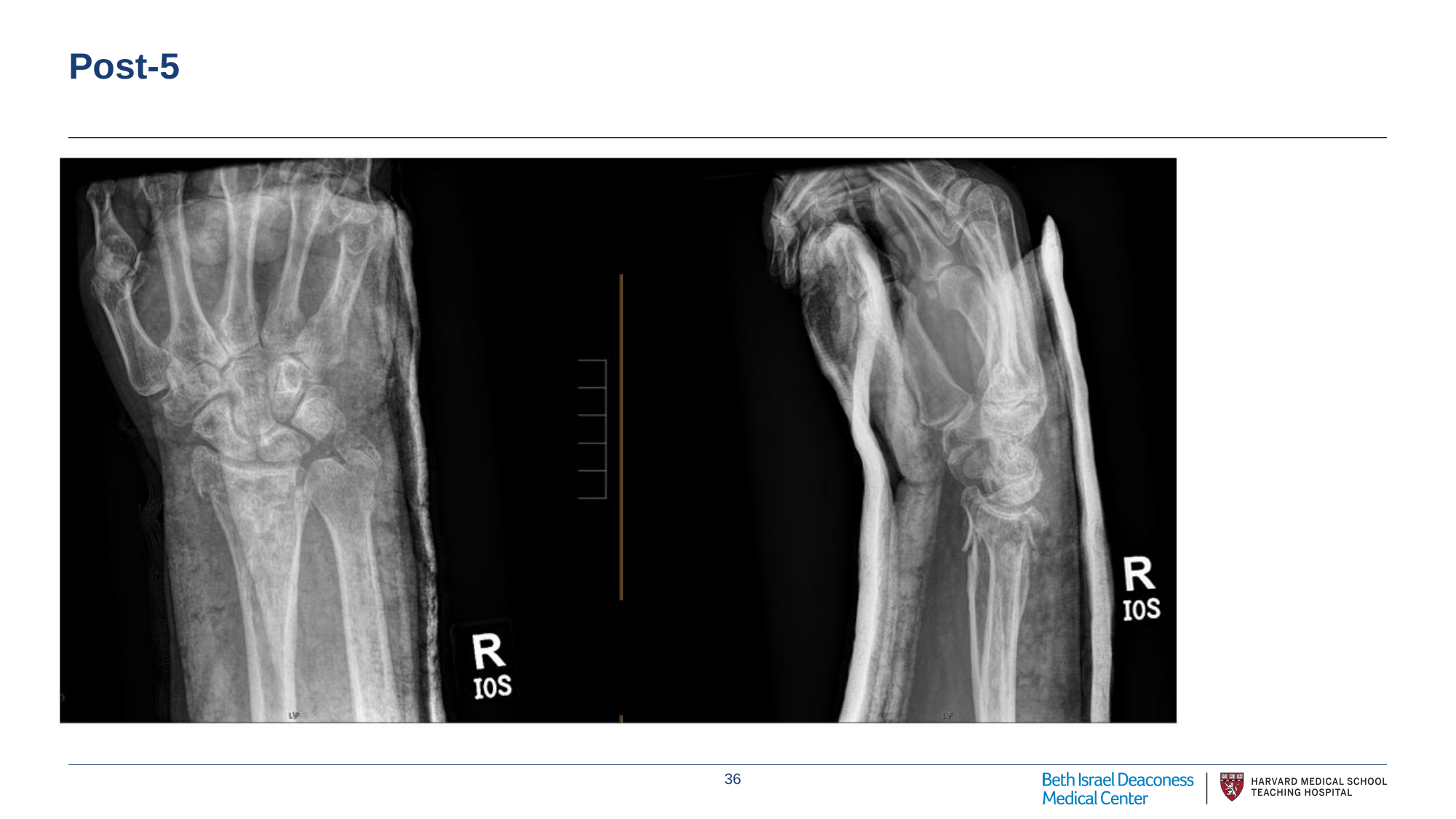

# Post-5
